# Evaluating Alkaline Phosphatase-Instructed Self-Assembly of d‑Peptide Diesters for Selectively Inhibiting Immunosuppressive Cancer Cells

**DOI:** 10.1021/acsomega.6c06074

**Published:** 2026-07-17

**Authors:** Meihui Yi, Gabriel Ashton-Rickardt, Yuchen Qiao, Yali Huang, Hongjian He, Bing Xu

**Affiliations:** † Department of Chemistry, 8244 Brandeis University, 415 South Street, Waltham, Massachusetts 02454, United States; ‡ Department of Electrical and Computer Engineering, Boston University, Boston, Massachusetts 02215, United States

## Abstract

Hypoxia-driven adenosinergic signaling suppresses antitumor immunity and enables many tumors to evade cancer immunotherapy. Alkaline phosphatase (ALP) contributes to this immunosuppression by hydrolyzing extracellular adenosine triphosphate (ATP) to adenosine. Rather than inhibiting this broadly essential enzyme, we exploit elevated ALP activity in tumors as a tumor-specific trigger for enzyme-instructed self-assembly (EISA). Using a potent naphthalene-capped phosphopeptide diester precursor (**1P**), which exhibits in vivo efficacy as a starting point, we designed and synthesized a series of analogs (**2P**–**15P**) that vary the N-terminal capping group and/or the position of the phosphotyrosine trigger to explore structure–activity relationships. We evaluated these analogs for anticancer activity in ALP-overexpressing cancer cells (e.g., Saos-2), using ALP-low cells (e.g., HEK-293) as controls. One analog (**6P**) exhibited potent, selective cytotoxicity, with submicromolar GI_50_ values (∼0.3 μM) in ALP-high cancer cells. Notably, **6P** demonstrated submicromolar GI_90_ values and surpassed the efficacy of cisplatin and paclitaxel against Saos-2 cells. These findings highlight the potential of rationally designed d-phosphopeptide diesters to enable ALP-responsive self-assembly and selectively inhibit the growth of ALP-overexpressing tumors. This strategy offers a promising platform for the development of supramolecular therapeutics targeting immunosuppressive tumor microenvironments.

## Graphical abstract

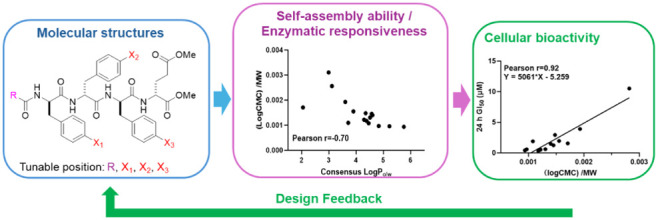

## Introduction

Cancer remains a formidable challenge to human health, often evading therapy through complex survival mechanisms.^1–4^ Despite the transformative progress of immunotherapy,^5–9^ hypoxia-driven adenosinergic signaling frequently suppresses antitumor immunity, allowing many tumors to escape immune surveillance.^10–13^ Alkaline phosphatase (ALP),^14^ an ectoenzyme overexpressed in various malignant tissues, significantly contributes to this immunosuppression by hydrolyzing extracellular adenosine triphosphate (ATP) into adenosine.^15–20^ While conventional strategies focus on inhibiting such enzymes,^21–23^ their effectiveness diminishes in the presence of ALP overexpression. On the other hand, the elevated levels of ALP within the tumor microenvironment (TME) present a compelling opportunity to exploit this enzyme as a selective trigger for therapeutic intervention.

Thus, instead of blocking its essential physiological functions, we and others harness ALP to trigger enzyme-instructed self-assembly (EISA).^24–52^ EISA uses ALP-mediated dephosphorylation as the initiating biochemical event to convert small, soluble peptide precursors into larger, nondiffusive supramolecular nanostructures in situ.^53^ The local formation of supramolecular assemblies on or near cancer cells perturbs cellular functions and compromises cell viability. Unlike traditional inhibitors, which lose efficacy as enzyme levels rise (amplification), EISA analogs become more potent as enzyme expression increases, turning a common drug-resistance mechanism into a therapeutic advantage.^54^ We have found that the naphthalene-capped phosphopeptide diester precursor Nap-ff_p_ye­(OMe)_2_ (**1P**) inhibits an ALP-overexpressing osteosarcoma (Saos-2 and Saos-2-lung) in cell assay and strongly suppress tumor growth in an orthotopic mouse model without harming normal organs.^55^ Importantly, the mechanism underlying enzyme-instructed peptide self-assembly is increasingly understood. Recent cryo-structural work has shown that alkaline-phosphatase-triggered peptide self-assembly can drive extrinsic lytic cell death,^53^ providing direct structural and mechanistic support for EISA-enabled pyroptosis for enhancing immunotherapy.^56^ In addition, earlier work on prion-like nanofibrils of small molecules demonstrated selective inhibition of cancer cells through disruption of cytoskeletal dynamics, establishing that supramolecular assemblies can exert anticancer activity through defined cellular mechanisms rather than nonspecific aggregation.^57^

In contrast to most studies that use ALP-based EISA for targeting various cancers through incorporation of additional therapeutic agents, the study of **1P** ^55^ represents the first case of EISA being used to target immunosuppressive tumors in vivo without other therapeutic agents.

Building upon the previous identification of a naphthalene-capped d-phosphopeptide diester (**1P**) with promising ALP-responsive self-assembly and anticancer properties arising from its response to both surface and intracellular ALP (Scheme 1),^58^ we sought to systematically investigate how subtle structural modifications influence assembly propensity, enzyme responsiveness, and the resulting bioactivity. To this end, we developed a series of analogs (**2P**–**15P**) by altering the N-terminal capping group and/or repositioning the phosphotyrosine trigger. These modifications aimed to fine-tune the EISA process and modulate interactions with ALP-overexpressing tumor cells for developing potent anticancer EISA agents.

**Scheme 1:**
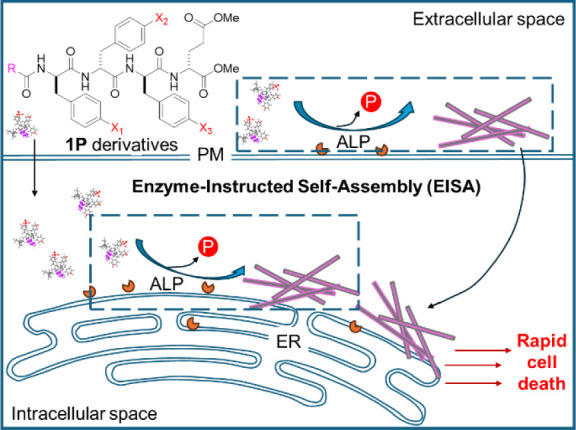
General Structures of the d-Peptide Diesters and EISA at the Plasma Membrane and ER for Inhibiting Immunosuppressive Cancer Cells

To evaluate the biological relevance of these structural variations, we conducted cytotoxicity assays across different cancer cell lines with varying ALP expressions, including osteosarcoma and ovarian carcinoma, as well as ALP-low control cell lines (HEK-293). This approach enabled us to assess both potency and selectivity linked to ALP activity. Among the analogs tested, one analog (**6P**) emerged as particularly effective, exhibiting strong antiproliferative effects with GI_50_ values in the submicromolar range (∼310 nM) in both ALP-high cell lines. Notably, this analog demonstrated GI_50_ values (0.3 μM) exceeding the efficacy of clinical chemotherapeutics such as cisplatin (41 μM)^59^ and paclitaxel (1.0 μM)^60^ against Saos-2 cells. In addition, for **2P**, cytotoxicity exhibited a strong correlation with ALPL expression across different cell lines. These results underscore the utility of molecular design in modulating ALP responsive supramolecular building blocks and support the development of d-phosphopeptide diesters as an effective class of tumor-selective agents. Collectively, our findings establish a structure–activity framework for engineering EISA-based therapeutics against ALP-overexpressing tumor cells. Although this study does not directly evaluate immune-related outcomes, the focus on ALP-high Saos-2 cells is biologically relevant because ALP participates in adenosinergic immunosuppression and our previous study showed that ALP-triggered EISA suppresses Saos-2 orthotopic bone tumor growth in vivo.^55^ Thus, the immunosuppression-related context provides motivation for this work, while direct immune modulation remains an important direction for future study.

## Results and Discussion

### Molecular Design and Synthesis

As shown in Scheme 2, the analogs were designed from the parent precursor **1P** ^55^ to systematically vary two key structural parameters—the N-terminal capping group and the position of the phosphotyrosine trigger—thereby enabling us to correlate aromaticity, hydrophobicity, and rigidity with self-assembly propensity, ALP-mediated responsiveness, and the resulting cell-selective bioactivity. The parent precursor, **1P**, has a basic structure consisting of an N-terminal capping group (R), a central d-triaromatic amino-acid motif, and a C-terminal d-glutamic acid dimethyl ester. Within the triaromatic motif, X_1_, X_2_, and X_3_ denote positions that can be either hydrogen or phosphate. To investigate the influence of N-terminal capping groups on biological activity, we varied R to generate precursors **2P** and **5P**–**15P**. In addition, while maintaining the biphenyl group as the N-terminal cap, we systematically relocated the phosphate from X_3_ to X_1_ or X_2_, yielding **3P** and **4P**, respectively. This design allows us to elucidate the role of phosphate position in regulating enzyme-instructed self-assembly and biological activity. In addition, to verify the role of the phosphate group, we removed the phosphate from **2P** to obtain compound **2**.

**Scheme 2:**
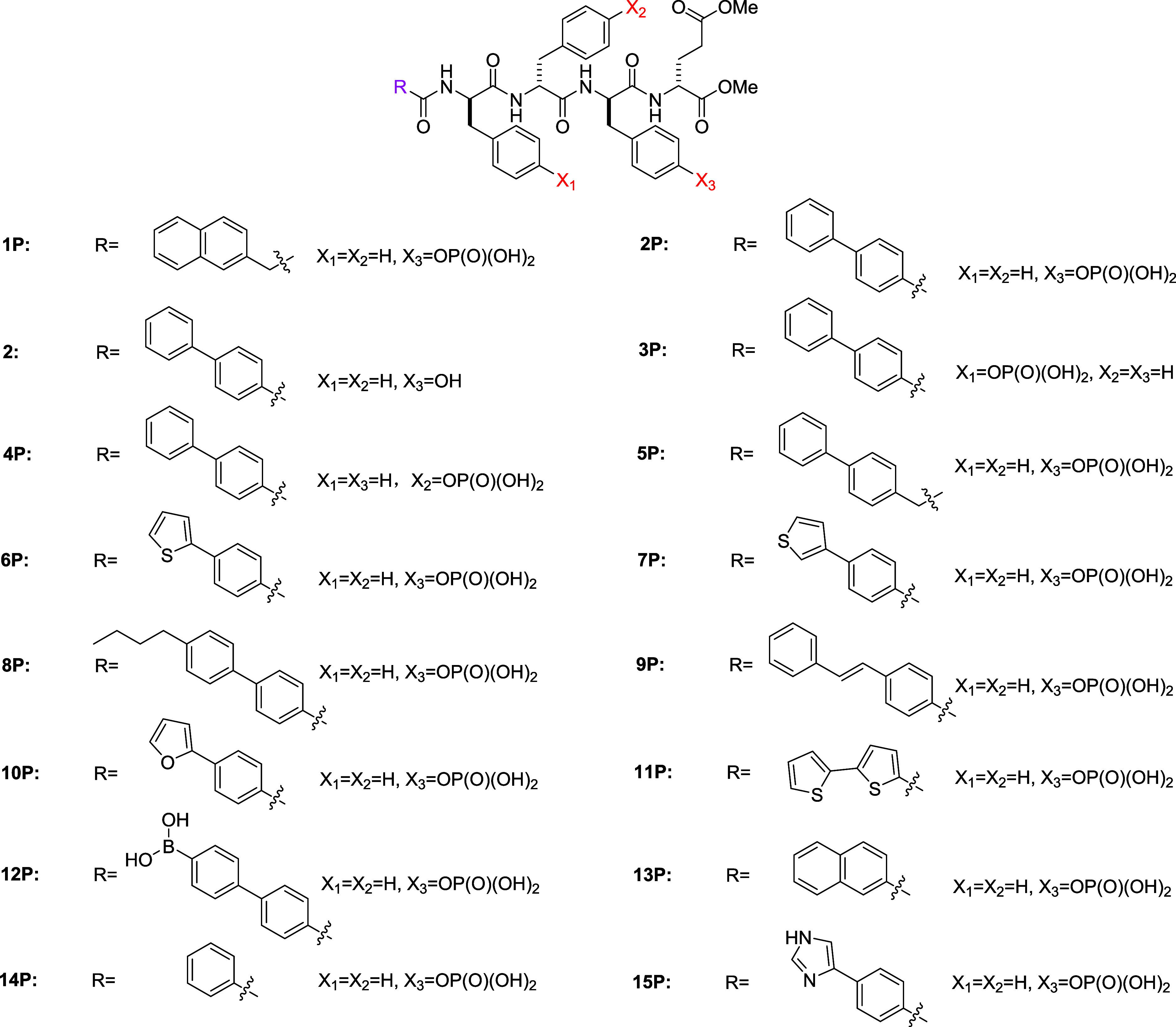
Molecular Structures of **1P**, and Analogs **2P**−**15P**

All peptides without methylation were synthesized by solid-phase peptide synthesis (SPPS) (Scheme S2). Briefly, Fmoc-protected amino acids were loaded onto 2-chlorotrityl chloride resin. After removal of the Fmoc protecting group with piperidine in N, N-dimethylformamide (DMF), subsequent amino acids or the N-terminal capping group were coupled sequentially to elongate or cap the peptide chain. The peptides were then cleaved from the resin using trifluoroacetic acid (TFA). To obtain methylated analogs,

the resulting peptides (1 equiv) were dissolved in dichloromethane under stirring, followed by the addition of bromotrimethylsilane (30 equiv). The reaction mixture was stirred at room temperature overnight. After removal of solvents by air drying, methanol was added, and the mixture was stirred at room temperature for an additional 24 h. Following purification by HPLC, we obtained analogs **2P**–**15P** in good yields with purity over 95% as shown in Figure S8–S23.

### Self-Assembly

First, we determined the critical micelle concentration (CMC) of these analogs using Rhodamine 6G fluorescence. As shown in Table 1 and Figure S1, **14P**, which contains a benzoic acid capping group, **1P** with a naphthylene group, and **2P**–**4P** bearing biphenyl motifs exhibited a progressive decrease in CMC from approximately 343.1 μM to 50 μM and further to 10–20 μM.

**Table 1:** Critical Micelle Concentration (CMC) and Log P_o/w_ of Analogs **1P**−**15P** ^a^

Analogues	CMC (μM)	Consensus Log P_o/w_
1P	50.0	3.60
2P	14.6	4.51
2	6.00	5.22
3P	17.8	4.60
4P	19.7	4.57
5P	21.9	4.35
6P	11.4	4.38
7P	12.4	4.29
8P	7.70	5.75
9P	7.74	4.83
10P	9.24	3.71
11P	9.43	4.46
12P	39.8	2.05
13P	22.1	3.90
14P	343.1	2.99
15P	182.6	3.11

a The Consensus Log P was Calculated Using SwissADME.

These results indicate a positive correlation between the number of aromatic units and self-assembly capability. To further tune hydrophobicity, a hydrophobic C^4^H^6^ chain or a hydrophilic boronic acid moiety was introduced onto the biphenyl capping group, yielding **8P** and **12P**, respectively. Consistent with increased hydrophobicity, **8P** displayed a markedly lower CMC (7.7 μM) than **12P** (39.8 μM), demonstrating that the self-assembly behavior can be modulated by incorporating hydrophobic or hydrophilic elements into the capping motif. Among **2P**, **3P**, and **4P**, which differ only in the position of the phosphate group, only minor variations in CMC were observed, suggesting that phosphate positioning has a limited effect on self-assembly. In contrast, comparison of **2P** with its dephosphorylated counterpart **2** revealed a substantial decrease in CMC upon phosphate removal, highlighting the enhanced self-assembly propensity induced by dephosphorylation.

Containing a biphenyl capping group but with an additional methylene spacer between the capping motif and the peptide backbone, **5P** exhibited a higher CMC, indicating increased conformational flexibility that disfavors assembly. Conversely, **13P**, bearing a naphthyl group with one fewer carbon linker compared to **1P**, showed a lower CMC, consistent with increased molecular rigidity. Notably, **9P**, which features a rigid olefinic linkage between the two phenyl rings, also exhibited a low CMC. Collectively, these results suggest an inverse relationship between molecular rigidity and CMC, where increased rigidity and aromaticity promote stronger self-assembly.

In addition, we explored the possibility of other heteroaromatic rings as capping groups to the peptides. **6P** contains a thiophene–phenyl conjugated capping group with 2-substituted thiophene, whereas **7P** is the corresponding regioisomer featuring 3-substituted thiophene. **10P** incorporates a furan aromatic capping group. **11P** bears a bithiophene group. These capping groups share a rigid, π-conjugated aromatic framework, composed of a phenyl ring conjugated with a five-membered heteroaromatic ring, and all shown very similar low CMC values. **15P** features an imidazole–phenyl capping group, introducing both aromaticity and hydrogen-bonding capability. The relatively large CMC value could be attributed to the deprotonation of imidazole, which makes it more hydrophilic compared to other heteroaromatic capping groups. To better visualize the relationship between CMC and hydrophobicity, the LogP was calculated using SwissADME and plotted with CMC and molecular weight. According to Figure 1, there is a clear negative correlation between CMC and LogP (Pearson *r* = −0.70), a common feature of amphiphilic molecules.^61^

**Figure 1:**
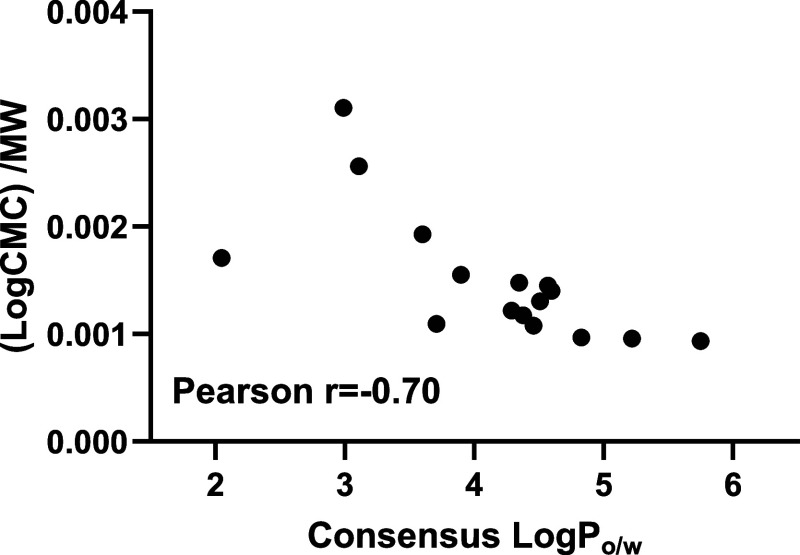
Relationship between CMC and logP of the analogs.

### Cell Inhibition

After determining the CMC values of these analogs, we next evaluated their cytotoxicity against different cell lines to better understand how molecular structure influences their biological activity. First, the importance of the phosphate group was confirmed by comparing analog **2P** with its dephosphorylated counterpart **2** (Figure 2a,b and S2a). In ALP overexpressing Saos-2 osteosarcoma cells, **2P** exhibited a 24 h GI_50_ of approximately 0.60 μM, whereas compound **2** showed a 24 h GI_50_ greater than 10 μM. Over 3 days, **2P** exhibited a dose-dependent response, whereas compound **2** showed no dose–response behavior. The substantial loss of activity upon removal of the phosphate group highlights its critical role as an enzymatic trigger for enzyme-instructed self-assembly (EISA). As shown in Figure S2b, 2P exhibited only mild effects on HEK-293 cells, which express ALP at normal levels; moreover, the decrease in HEK-293 viability observed after 72 h was not dose-responsive, suggesting that it is unlikely to result from a specific ALP-dependent EISA mechanism and may instead reflect nonspecific long-term culture or compound-exposure effects.

**Figure 2:**
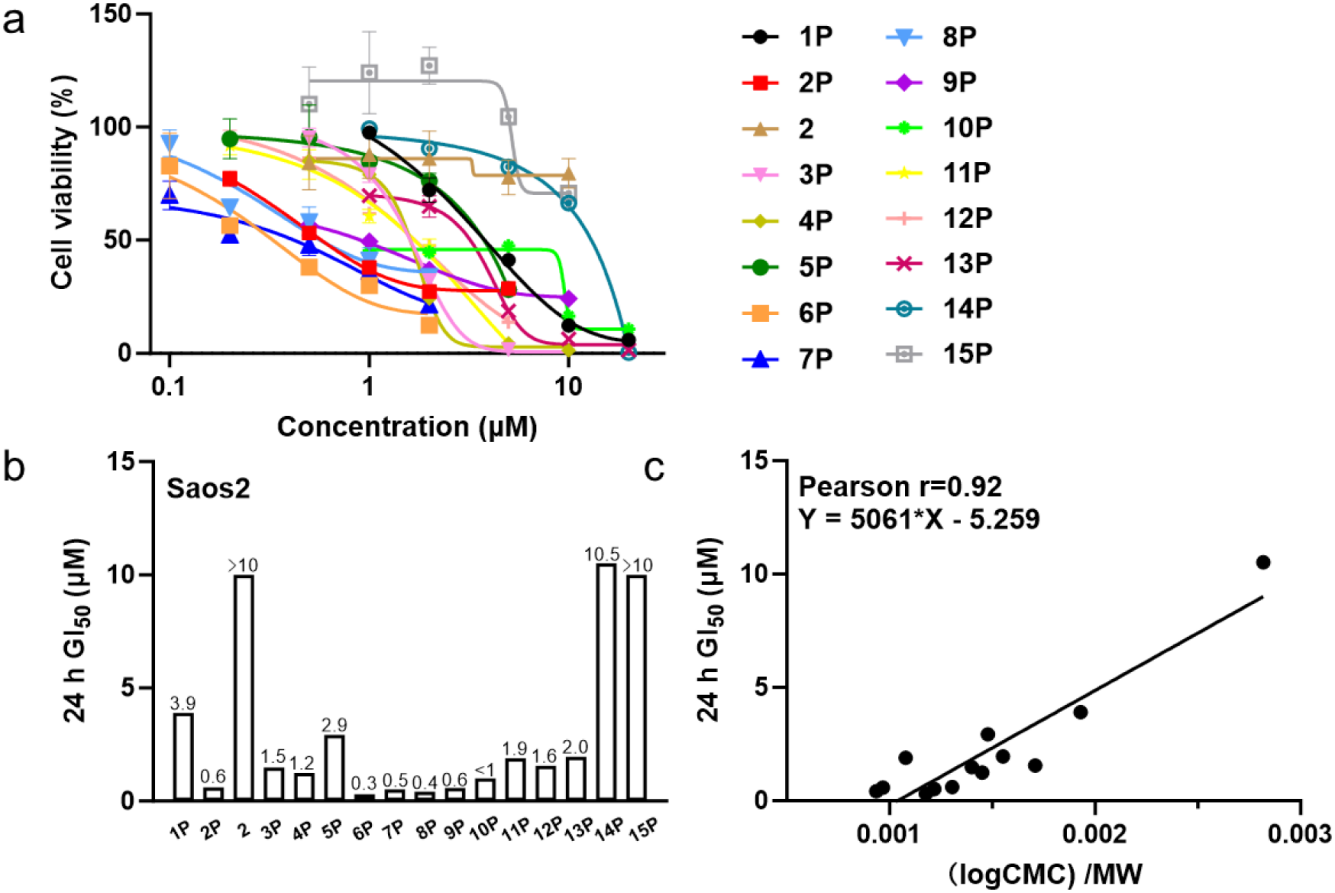
Cellular inhibition of **1P**−**15P**. a. The cell viability of Saos-2 cells treated with **1P**−**15P** for 24 h. b. The GI_50_ of **1P**−**15P** against Saos-2 cells for 24 h. c. The correlation between 24 h GI_50_ of analogs and their CMC.

Additionally, treatment with the tissue nonspecific alkaline phosphatase (ALPL) inhibitor 2,5-dimethoxy-*N*-(quinolin-3-yl) benzenesulfonamide (DQB) rescued Saos-2 cells from **2P** induced cytotoxicity in a dose dependent manner (Figure S2c). Together, these results support the essential role of the phosphate group and ALP triggered EISA in mediating the observed biological activity. The markedly lower activity of the dephosphorylated compound **2** compared with its phosphorylated precursor **2P** further confirms that **2P** does not act as a conventional prodrug releasing **2**, but instead requires the phosphate group as an ALP-responsive trigger for productive EISA and cytotoxicity.

While retaining the phosphate group near the C-terminus, we investigated the influence of N-terminal capping groups on cellular activity using Saos-2 cells. Among the analogs, **6P** exhibited the highest cytotoxicity toward Saos-2 cells, with a 24 h GI_50_ of approximately 0.31 μM. Notably, **2P**, **6P**, **7P**, **8P**, and **9P** displayed similar GI_50_ values in the submicromolar range, consistent with their comparable CMC values. In contrast, **1P**, **14P**, and **15P**, which possess higher CMC values, showed reduced cytotoxicity toward Saos-2 cells. The trends observed over the 3 days were similar, as shown in Figure S3. To further examine this relationship, the correlation between cytotoxicity (GI_50_) and CMC was analyzed (Figure 2c), revealing a strong positive linear correlation between CMC and cellular activity (Pearson *r* = 0.92). Given that lower CMC values indicate stronger self-assembly propensity, these results further confirm that increased cytotoxicity of EISA arises from the enhanced self-assembly ability of the peptide building blocks.^58^

Among the low-CMC analogues, **6P** exhibited the strongest cytotoxicity toward Saos-2 cells, suggesting that CMC alone does not fully account for cellular activity. The enhanced activity of **6P** may arise from the thiophene-containing N-terminal capping group, which provides a rigid, π-conjugated, and moderately hydrophobic motif that favors productive supramolecular assembly after ALP-mediated dephosphorylation. Compared with other low-CMC analogues, such as **7P**, **8P**, and **9P**, the 2-substituted thiophene–phenyl cap in **6P** may better balance hydrophobicity, aromatic stacking, and molecular geometry, thereby promoting the formation of more effective assemblies in the cellular environment. Consistent with this possibility, TEM (Figure 4) showed that **6P** formed fiber-like structures even before ALP treatment and generated more extensive, well-defined fibrous networks after ALP treatment. Although these TEM results do not establish a direct structure–activity relationship, they suggest that the thiophene-containing cap of **6P** may enhance ALP-responsive assembly and/or interactions of the resulting assemblies with Saos-2 cells. Thus, the superior potency of **6P** likely reflects the combined effects of low CMC, favorable heteroaromatic capping, efficient enzyme responsiveness, and productive cellular interactions.

In addition, the position of the phosphate group within the peptide sequence appears to be an important determinant of activity. Notably, the cellular responses to **2P**, **3P**, and **4P** varied across different cell lines (Figures 3a and S5–S7). For **2P**, in which the phosphate group is located distal to the aromatic capping group, two distinct response patterns were observed, with some cell lines exhibiting clear dose–response behavior while others showed minimal or no response. In contrast, **3P**, where the phosphate group is positioned proximal to the aromatic capping group, consistently displayed dose–response behavior across all tested cell lines, albeit with varying sensitivity. Notably, **4P**, in which the phosphate group is located between these two cases, exhibited intermediate behavior, with cellular responses falling between those observed for **2P** and **3P**.

**Figure 3:**
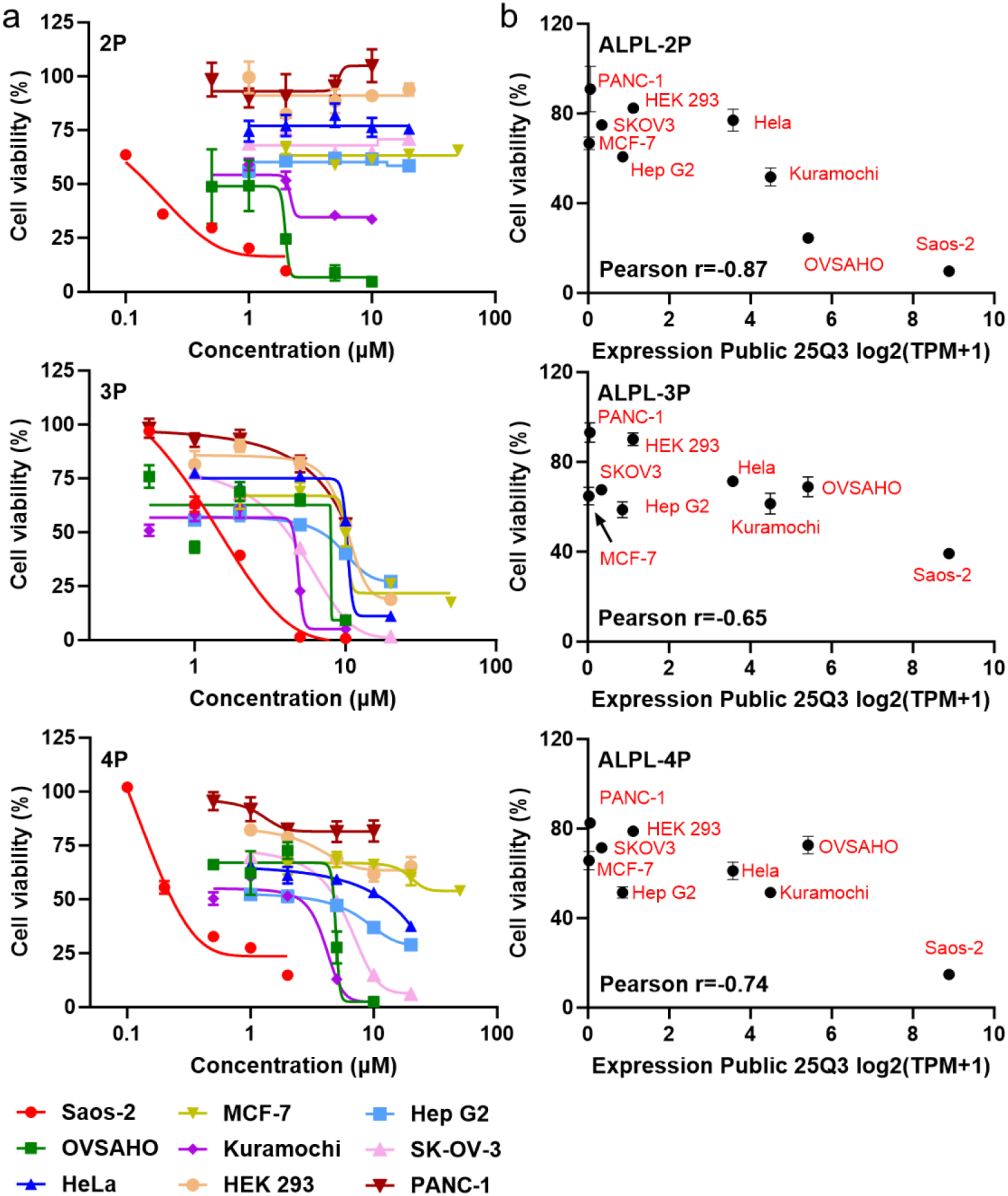
Relationship between gene expression and cell inhibition. a. The cell viability of different cell lines being treated with **2P**, **3P**, and **4P** for 24 h. b. The cell viability of different cell lines at 2 μM for 24 h versus ALPL gene expression levels according to Depmap CCLE.

To better understand this relationship, we examined DepMap CCLE Cell Line Gene Expression Profiles for ALPL, ALPP, ALPP2, and ALPI. As shown in Figure 3b, **2P** showed the strongest relationship between gene expression of ALPL and cell viability (Pearson *r* = – 0.87). For **3P** and **4P**, the correlation between ALPL expression and cytotoxicity was moderate (Pearson r = – 0.65 and −0.74, respectively), suggesting that additional factors, including other alkaline phosphatases, alternative enzymes, and distinct cellular pathways, may be involved. To further understand the contribution of other alkaline phosphatases, we analyzed the expression of ALPP, ALPP2, and ALPI. However, as shown in Figure S4, the correlations between their gene expression levels and cell viability were weak, with Pearson r values below 0.5. These results suggest that the spatial positioning of the phosphate group relative to the aromatic capping moiety plays a critical role in modulating peptide activity, potentially by influencing enzymatic accessibility and subsequent self-assembly.

### Enzyme-Instructed Self-Assembly

Based on the correlations observed above, peptide self-assembly is likely involved in modulating cellular activity. To gain preliminary insight into the morphology of the assemblies, transmission electron microscopy (TEM) was performed on several key precursors. As shown in Figure 4a, three derivatives (**1P**, **6P**, and **8P**) that differ only in capping groups exhibit distinct self-assembly behaviors, both before and after enzymatic treatment. At 10 μM, **1P**, which has a relatively high CMC, primarily forms aggregates and shows little morphological change upon ALP treatment. In contrast, **6P** and **8P**, with CMC values below 10 μM, are capable of forming fiber-like structures even in the absence of enzyme. Upon ALP treatment, more extensive and well-defined fibrous networks are observed for these compounds. Three additional derivatives (**2P**, **3P**, and **4P**), which differ in the position of the phosphate group, were further examined. As their CMC values fall within the range of 10–20 μM, a concentration of 20 μM was selected for TEM analysis. As shown in Figure 4b, despite their similar CMC values, these compounds exhibit markedly different assembly behaviors. At this concentration, **2P** undergoes a morphological transition from aggregates to fibrous structures upon enzymatic treatment. In contrast, **3P** maintains a similar morphology before and after enzyme treatment, indicating limited structural change. Notably, **4P** is capable of forming fibrous structures even in the absence of enzyme, and its morphology shows minimal change upon enzymatic treatment. These results demonstrate that peptide self-assembly is strongly influenced by both the capping group and the position of the phosphate group, which together determine assembly behavior and responsiveness to enzymatic treatment.

**Figure 4:**
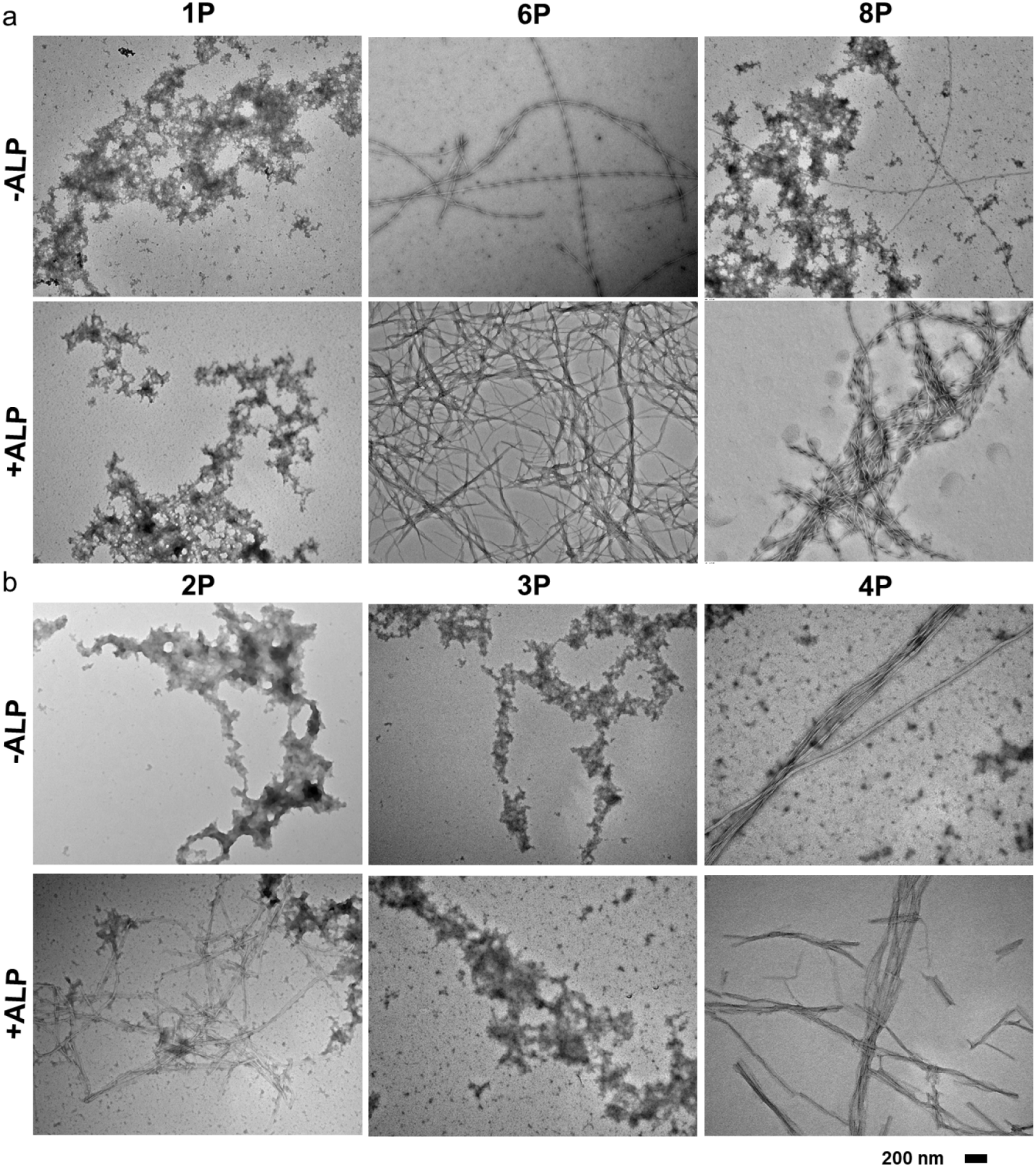
Enzyme-instructed self-assembly of peptide derivatives. a. The TEM images of **1P**, **6P**, and **8P** at 10 μM with or without the treatment with 1 U/mL ALP for 24 h. b. The TEM images of **2P**, **3P**, and **4P** at 20 μM with or without the treatment of 1 U/mL ALP for 24 h.

While TEM provides preliminary insight into the morphology of the assemblies, the structural information remains limited and precludes direct correlation of TEM with cellular activity. Therefore, more advanced techniques, such as cryo-electron microscopy (cryo-EM) or cryo-electron tomography (cryo-ET), which enable direct visualization of peptide assemblies in cellular environments,^53^ are required to elucidate the relationship between supramolecular structure and biological function.

## Conclusion

In summary, this study establishes that the anticancer activity of ALP-responsive d-peptide diesters is governed by two coupled design parameters: the propensity of the precursors to assemble and the accessibility of the phosphotyrosine motif to enzymatic dephosphorylation. By systematically varying the N-terminal capping groups and the position of the phosphate trigger, we found that changes in aromaticity, hydrophobicity, and molecular rigidity alter CMC and thereby tune assembly propensity, which strongly correlates with cellular activity. At the same time, shifting the phosphotyrosine closer to the aromatic cap diminishes the relationship between activity and ALPL expression, indicating that enzyme accessibility is an equally important determinant of productive EISA. Together, these results show that potent ALP-triggered bioactivity arises from a balance between efficient enzymatic conversion and a strong tendency of the resulting molecules to assemble in situ.

More broadly, these findings provide a practical framework for designing EISA analogs for ALP-overexpressing tumors: effective precursors should combine low-CMC scaffolds with phosphate placements that remain readily processed by ALP in cellular environments. Within this framework, the exceptional potency of **6P** is especially informative. Because **6P**, which bears a heteroaromatic thiophene-containing N-terminal cap, emerged as the most active and selective analog, heteroaromatic N-terminal groups appear to be a particularly promising design space for further optimization. Moreover, the trend exhibited by the N-terminal capping groups may provide a reference point for other EISA based anticancer therapeutics.^62–64^ Future studies should therefore expand this motif class systematically, for example by varying heteroaromatic identity, substitution pattern, electronic properties, and ring connectivity, to determine whether the strong activity of **6P** reflects a broader advantage of heteroaromatic capping groups in promoting favorable enzyme responsiveness and supramolecular assembly. Nevertheless, further in vivo validation, pharmacokinetic analysis, and comprehensive toxicity evaluation will be required to establish **6P** as a viable lead analog for therapeutic development.

Although the present study focuses on the cellular structure–activity relationship of these ALP-responsive d-peptide diesters, our previous in vivo work^55^ with the related precursor **1P** demonstrated effective inhibition of bone tumor growth without obvious systemic toxicity, supporting the feasibility of this EISA strategy in vivo while highlighting the need for future studies to directly quantify hydrolysis kinetics, CMC-dependent pharmacokinetic effects, tissue retention, and biological clearance.

## Supplementary Material



## Notes

Detailed experimental procedures and materials; LC/MS results of individual compounds; additional cell viability data (PDF)

## #

(M.Y.) Laboratory for Biomaterials and Drug Delivery, Department of Anesthesiology, Division of Critical Care Medicine, Boston Children’s Hospital, Harvard Medical School, Boston, MA 02115, United States

## ∇

(G.A.-B.) Division of Chemistry and Chemical Engineering, California Institute of Technology

## Abbreviations

ALP: alkaline phosphatase
EISA: enzyme-instructed peptide self-assembly
ff: di-d-phenylalanine
CMC: critical micelle concentration
TEM: transmission electron microscopy

## References

[1] Siegel R. L., Kratzer T. B., Wagle N. S., Sung H., Jemal A. (2026). Cancer statistics,
2026. Ca-Cancer J. Clin..

[2] Hanahan D. (2022). Hallmarks
of Cancer: New Dimensions. Cancer Discovery.

[3] Hanahan D., Weinberg R. A. (2011). Hallmarks of cancer: The next generation. Cell.

[4] Dagogo-Jack I., Shaw A. T. (2018). Tumour heterogeneity and resistance
to cancer therapies. Nat. Rev. Clin. Oncol..

[5] Fridman W. H., Pagès F., Saut̀s-Fridman C., Galon J. (2012). The immune
contexture in human tumours: Impact on clinical outcome. Nat. Rev. Cancer.

[6] Pardoll D. M. (2012). The blockade
of immune checkpoints in cancer immunotherapy. Nat. Rev. Cancer.

[7] Dong H., Strome S. E., Salomão D. R., Tamura H., Hirano F., Flies D. B., Roche P. C., Lu J., Zhu G., Tamada K. (2002). Tumor-associated B7-H1 promotes T-cell apoptosis: A
potential mechanism of immune evasion. Nat.
Med..

[8] Vesely M. D., Zhang T., Chen L. (2022). Resistance Mechanisms to Anti-PD
Cancer Immunotherapy. Annu. Rev. Immunol..

[9] Yang X., Chen L. (2025). Why has immune “checkpoint”
therapy failed in most
clinical trials?. J. Immunother Cancer.

[10] Sitkovsky M., Ohta A. (2013). Targeting the hypoxia-adenosinergic
signaling pathway to improve
the adoptive immunotherapy of cancer. J. Mol.
Med..

[11] Wang L., Zhang J., Zhang W., Zheng M., Guo H., Pan X., Li W., Yang B., Ding L. (2024). The inhibitory
effect
of adenosine on tumor adaptive immunity and intervention strategies. Acta Pharm. Sin. B.

[12] Fong L., Hotson A., Powderly J. D., Sznol M., Heist R. S., Choueiri T. K., George S., Hughes B. G. M., Hellmann M. D., Shepard D. R. (2020). Adenosine
2A Receptor Blockade as an Immunotherapy
for Treatment-Refractory Renal Cell Cancer. Cancer Discovery.

[13] Sun C., Wang B., Hao S. (2022). Adenosine-A2A Receptor Pathway in
Cancer Immunotherapy. Front. Immunol..

[14] Simopoulos T. T., Jencks W. P. (1994). Alkaline phosphatase
is an almost perfect enzyme. Biochemistry.

[15] Vigano S., Alatzoglou D., Irving M., Ménétrier-Caux C., Caux C., Romero P., Coukos G. (2019). Targeting Adenosine
in Cancer Immunotherapy to Enhance T-Cell Function. Front. Immunol..

[16] Yegutkin G. G., Boison D. (2022). ATP and Adenosine Metabolism
in Cancer: Exploitation
for Therapeutic Gain. Pharmacol. Rev..

[17] Millán J. L. (2006). Alkaline
Phosphatases. Purinergic Signalling.

[18] Picher M., Burch L. H., Hirsh A. J., Spychala J., Boucher R. C. (2003). Ecto 5′-Nucleotidase
and Nonspecific Alkaline Phosphatase: Two Amp-hydrolyzing Ectoenzymes
With Distinct Roles In Human Airways*. J. Biol.
Chem..

[19] Pickkers P., Snellen F., Rogiers P., Bakker J., Jorens P., Meulenbelt J., Spapen H., Tulleken J. E., Lins R., Ramael S. (2009). Clinical pharmacology of exogenously administered alkaline
phosphatase. Eur. J. Clin. Pharmacol..

[20] Rader B. A. (2017). Alkaline
Phosphatase, an Unconventional Immune Protein. Front. Immunol..

[21] Bai Y., Zhang X., Zheng J., Liu Z., Yang Z., Zhang X. (2022). Overcoming high level adenosine-mediated
immunosuppression by DZD2269,
a potent and selective A2aR antagonist. J. Exp.
Clin. Cancer Res.

[22] Thompson E. A., Powell J. D. (2021). Inhibition of the
Adenosine Pathway to Potentiate Cancer
Immunotherapy: Potential for Combinatorial Approaches. Annu. Rev. Med..

[23] Wang Y., Hu X., Pandey S., Khatri U., Shen T., Subbiah V., Mooers B. H. M., Chao T., Wang S., Yu H. (2025). Targeting
Oncogenic RET Kinase by Simultaneously Inhibiting Kinase
Activity and Degrading the Protein. J. Med.
Chem..

[24] Yang Z., Gu H., Fu D., Gao P., Lam J. K., Xu B. (2004). Enzymatic
Formation of Supramolecular Hydrogels. Adv.
Mater..

[25] Yang Z., Xu B. (2007). Supramolecular hydrogels based on biofunctional nanofibers of self-assembled
small molecules. J. Mater. Chem..

[26] Zhou J., Xu B. (2015). Enzyme-instructed self-assembly:
A multistep process for potential
cancer therapy. Bioconjugate Chem..

[27] Toledano S., Williams R. J., Jayawarna V., Ulijn R. V. (2006). Enzyme-triggered
self-assembly of peptide hydrogels via reversed hydrolysis. J. Am. Chem. Soc..

[28] Zhang H., Wang Z., Gao T., Wang Z., Ren C., Liu J. (2023). An enzyme-instructed
self-assembly system induces tumor acidosis
via sequential-dual effect for cancer selective therapy. Acta Biomater..

[29] Gan S., Yang L., Heng Y., Chen Q., Wang D., Zhang J., Wei W., Liu Z., Njoku D. I., Chen J. L. (2024). Enzyme-Directed and Organelle-Specific Sphere-to-Fiber
Nanotransformation Enhances Photodynamic Therapy in Cancer Cells. Small Methods.

[30] Xie L., Ding Y., Zhang X., Zhang Z., Zeng S., Wang L., Yang Z., Liu Q., Hu Z. -W. (2023). A Cascade-Targeted
Enzyme-Instructed Peptide Self-Assembly Strategy for Cancer Immunotherapy
through Boosting Immunogenic Cell Death. Small
Methods.

[31] Yi M., Wang F., Tan W., Hsieh J. T., Egelman E. H., Xu B. (2022). Enzyme Responsive Rigid-Rod
Aromatics Target “Undruggable”
Phosphatases to Kill Cancer Cells in a Mimetic Bone Microenvironment. J. Am. Chem. Soc..

[32] Yi M., Feng Z., He H., Dinulescu D., Xu B. (2023). Evaluating Alkaline Phosphatase-Instructed
Self-Assembly of d-Peptides
for Selectively Inhibiting Ovarian Cancer Cells. J. Med. Chem..

[33] Yi M., Ashton-Rickardt G., Tan W., Liu Z., He H., Hsieh J. T., Xu B. (2024). Accelerating
Cellular Uptake with
Unnatural Amino Acid for Inhibiting Immunosuppressive Cancer Cells. Chemistry.

[34] Tan W., Zhang Q., Hong P., Xu B. (2022). A Self-Assembling Probe
for Imaging the States of Golgi Apparatus in Live Single Cells. Bioconjugate Chem..

[35] Feng Z., Wang H., Wang S., Zhang Q., Zhang X., Rodal A. A., Xu B. (2018). Enzymatic
Assemblies Disrupt the
Membrane and Target Endoplasmic Reticulum for Selective Cancer Cell
Death. J. Am. Chem. Soc..

[36] Feng Z., Wang H., Zhou R., Li J., Xu B. (2017). Enzyme-Instructed
Assembly and Disassembly Processes for Targeting Downregulation in
Cancer Cells. J. Am. Chem. Soc..

[37] Du X., Zhou J., Wang H., Shi J., Kuang Y., Zeng W., Yang Z., Xu B. (2017). In situ generated D-peptidic
nanofibrils as multifaceted apoptotic inducers to target cancer cells. Cell Death Dis.

[38] Zhou J., Du X., Yamagata N., Xu B. (2016). Enzyme-Instructed Self-Assembly of
Small D-Peptides as a Multiple-Step Process for Selectively Killing
Cancer Cells. J. Am. Chem. Soc..

[39] Zhou J., Du X., Xu B. (2016). Regulating the Rate of Molecular Self-Assembly for
Targeting Cancer Cells. Angew. Chem., Int. Ed..

[40] Gao Y., Kuang Y., Guo Z. F., Guo Z., Krauss I. J., Xu B. (2009). Enzyme-instructed
molecular self-assembly confers nanofibers and
a supramolecular hydrogel of taxol derivative. J. Am. Chem. Soc..

[41] Wang H., Feng Z., Wang Y., Zhou R., Yang Z., Xu B. (2016). Integrating Enzymatic
Self-Assembly and Mitochondria Targeting for
Selectively Killing Cancer Cells without Acquired Drug Resistance. J. Am. Chem. Soc..

[42] Wen X., Zhang R., Hu Y., Wu L., Bai H., Song D., Wang Y., An R., Weng J., Zhang S. (2023). Controlled sequential
in situ self-assembly and disassembly
of a fluorogenic cisplatin prodrug for cancer theranostics. Nat. Commun..

[43] Liu J., Li R. S., Zhang L., Wang J., Dong Q., Xu Z., Kang Y., Xue P. (2023). Enzyme-Activatable Polypeptide for
Plasma Membrane Disruption and Antitumor Immunity Elicitation. Small.

[44] Fan P., Guan Y., Zhang X., Wang J., Xu Y., Song B., Zhang S., Wang H., Liu Y., Qiao Z. Y. (2023). Cell membrane-specific
self-assembly of peptide nanomedicine
induces tumor immunogenic death to enhance cancer therapy. Nanoscale Horiz..

[45] Wang C., Zheng Y., Zheng Y., Wu C., Wang X., Huang M., Wu X., Zhong W., Xu K. (2022). Enzymatic
Synthesis of Peptide Nanofibers for Self-Delivery of Indomethacin
and Tyroservatide in Cancer Therapy. ACS Biomater.
Sci. Eng..

[46] Cheng X., Xia T., Zhan W., Xu H. D., Jiang J., Liu X., Sun X., Wu F. G., Liang G. (2022). Enzymatic Nanosphere-to-Nanofiber
Transition and Autophagy Inducer Release Promote Tumor Chemotherapy. Adv. Healthcare Mater..

[47] Zheng D., Liu J., Ding Y., Xie L., Zhang Y., Chen Y., Peng R., Cai M., Wang L., Wang H. (2021). Tandem molecular self-assembly
for selective lung cancer therapy
with an increase in efficiency by two orders of magnitude. Nanoscale.

[48] Gao Y., Gao J., Mu G., Zhang Y., Huang F., Zhang W., Ren C., Yang C., Liu J. (2020). Selectively enhancing radiosensitivity
of cancer cells via in situ enzyme-instructed peptide self-assembly. Acta Pharm. Sin. B.

[49] Gao Y., Zhang C., Chang J., Yang C., Liu J., Fan S., Ren C. (2019). Enzyme-instructed
self-assembly of a novel histone
deacetylase inhibitor with enhanced selectivity and anticancer efficiency. Biomater. Sci..

[50] Xu W., Feng R., Xu S., Wang L. (2026). Enzyme Catalysis Induced
Nanocluster Assembly into Micrometer-Size Monolayered Nanosheets with
Enhanced Near-Infrared Region II Emission. J.
Am. Chem. Soc..

[51] Du X., Zhou J., Wu L., Sun S., Xu B. (2014). Enzymatic
transformation of phosphate decorated magnetic nanoparticles for selectively
sorting and inhibiting cancer cells. Bioconjugate
Chem..

[52] Ontani A., Runser J.-Y., More S. H., Schmutz M., Chaumont A., Schroder A., Schaaf P., Jierry L. (2026). Features and mechanism
of localized enzyme-assisted self-assembly of peptides from unilamellar
vesicles. Front. Chem..

[53] Yi M., Guo J., Zia A., Guo W., Tachiyama S., Ashton-Rickardt G., Tan W., Qiao Y., Gong Y., Egelman E. H. (2026). Cryo-Structural
Insights into Enzymatic Peptide
Self-Assembly Driving Extrinsic Lytic Cell Death. J. Am. Chem. Soc..

[54] Zou L., Yi M., Xu B. (2025). Recent Advances
of In Situ Anticancer Nanomedicine
from Enzyme-Instructed Self-Assembly. ChemNanomat.

[55] Feng Z., Han X., Wang H., Tang T., Xu B. (2019). Enzyme-Instructed Peptide
Assemblies Selectively Inhibit Bone Tumors. Chem.

[56] Zhang J., Hu Y., Wen X., Yang Z., Wang Z., Feng Z., Bai H., Xue Q., Miao Y., Tian T. (2025). Tandem-controlled
lysosomal assembly of nanofibres induces pyroptosis for cancer immunotherapy. Nat. Nanotechnol..

[57] Kuang Y., Long M. J. C., Zhou J., Shi J., Gao Y., Xu C., Hedstrom L., Xu B. (2014). Prion-like
Nanofibrils of Small Molecules
(PriSM) Selectively Inhibit Cancer Cells by Impeding Cytoskeleton
Dynamics*. J. Biol. Chem..

[58] Feng Z., Wang H., Chen X., Xu B. (2017). Self-Assembling Ability
Determines the Activity of Enzyme-Instructed Self-Assembly for Inhibiting
Cancer Cells. J. Am. Chem. Soc..

[59] Zhang G., Li M., Zhu X., Bai Y., Yang C. (2011). Knockdown of Akt Sensitizes
Osteosarcoma Cells to Apoptosis Induced by Cisplatin Treatment. Int. J. Mol. Sci..

[60] Pucci B., Bellincampi L., Tafani M., Masciullo V., Melino G., Giordano A. (1999). Paclitaxel
Induces Apoptosis in Saos-2
Cells with CD95L Upregulation and Bcl-2 Phosphorylation. Exp. Cell Res..

[61] Israelachvili J. N., Mitchell D. J., Ninham B. W. (1976). Theory
of self-assembly of hydrocarbon
amphiphiles into micelles and bilayers. J. Chem.
Soc., Faraday Trans..

[62] Kalafatovic D., Nobis M., Son J., Anderson K. I., Ulijn R. V. (2016). MMP-9 triggered
self-assembly of doxorubicin nanofiber depots halts tumor growth. Biomaterials.

[63] Tan W., Zhang Q., Liu Z., Qiu K., Mahajan D., Gerton T., Copperman N., Dresselhaus E. C., Xia C., Lin C. (2026). Cycling
molecular assemblies for Golgi imaging
and disruption. Nat. Commun..

[64] Tan W., Zhang Q., Wang J., Yi M., He H., Xu B. (2021). Enzymatic
Assemblies of Thiophosphopeptides Instantly Target Golgi
Apparatus and Selectively Kill Cancer Cells*. Angew. Chem., Int. Ed..

