# Supplementary material for: Evaluating Alkaline Phosphatase-Instructed Self-Assembly of d‑Peptide Diesters for Selectively Inhibiting Immunosuppressive Cancer Cells
Source: ACS Omega. 2026 Jul 17;11(29):44506–14. doi: 10.1021/acsomega.6c06074 (PMC13425513; doi:10.1021/acsomega.6c06074)
Supplement: Supplementary file 1 [file ao6c06074_si_001.pdf]

Supplementary Information for

Evaluating Alkaline Phosphatase-Instructed Self-Assembly of D-Peptide Diesters for Selectively Inhibiting Immunosuppressive Cancer Cells

Meihui Yi, Gabriel Ashton-Rickardt, Yuchen Qiao, Yali Huang, Hongjian He, Bing Xu *

Department of Chemistry, Brandeis University, 415 South Street, Waltham, Massachusetts 02454, United States

Table of Contents

Experiment materials and instruments **2**
- Materials 2
- Instruments 2

Chemical synthesis **2**
- Synthesis of Fmoc-yp 2
- Synthesis of peptide derivatives (SPPS) 3
- Synthesis of methylated peptide derivatives 4

TEM sample preparation **4**
Critical micelle concentration (CMC) measurement **5**
Cell culture **5**
MTT assay **5**
Supplementary Figures **7**

Experiment materials and instruments

Materials

Fmoc protected amino acids, Fmoc-Osu, 2-Cl-trityl chloride resin (1.0-1.2 mmol/g) and HBTU were obtained from GL Biochem (Shanghai, China). N, N-diisopropylethylamine (DIPEA) and other chemical reagents and solvents were obtained from Fisher Scientific. Biphenyl-4-carboxylic acid, bromotrimethylsilane, 4-Biphenylacetic acid and benzoic acid was obtained from Sigma-Aldrich. 4-(Thiophen-2-yl)benzoic acid and 4-(Thiophen-3-yl)benzoic acid, [2,2′-Bithiophene]-5-carboxylic acid, 4'-Borono-[1,1'-biphenyl]-4-carboxylic acid, 2-Naphthoic acid and 4'-Butyl-[1,1'-biphenyl]-4-carboxylic acid were obtained from AmBeed. 4-[(1E)-2-Phenylethenyl]benzoic acid, 4-(2-Furyl)benzoic acid and 4-(1H-Imidazol-4-yl)benzoic acid was purchased from A2B. Methylthiazolyldiphenyl-tetrazolium bromide was purchased from ACROS Organics. Alkaline phosphatase was gotten from Biomatik (Cat. No. A1130, alkaline phosphatase [ALP], >1300 U/mg, in 50% glycerol.). Dulbecco's Modified Eagle Medium (DMEM), McCoy's 5A Medium, Minimum Essential Media (MEM), and RPMI-1640 Medium were purchased from ATCC. Fetal bovine serum (FBS) and Penicillin-Streptomycin from Gibco by Life Technologies. Reagents and solvents were used as received from commercial sources.

Instruments

All crude precursors and compounds were purified by a reverse phase HPLC (Agilent 1100 Series) equipped with an XTerra C18 RP column, and HPLC grade acetonitrile (0.1% TFA) and HPLC grade water (0.1% TFA) were used as the eluents. Part of LC-MS spectra were obtained on a Bruker timsTOF Pro Mass Spectrometer equipped with an Elute UHPLC chromatograph. And part of the LC-MS spectra were obtained with a Waters Acquity Ultra Performance LC with Waters MICROMASS detector. TEM images were taken on a Morgagni 268 transmission electron microscope. The absorbance of each well at 595 nm was measured by a DTX880 Multimode Detector.

Chemical synthesis

Synthesis of Fmoc-$_p$y

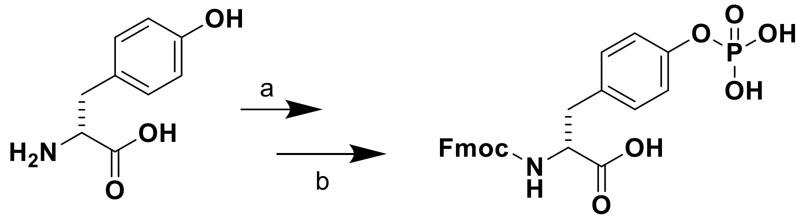

Scheme S1. The synthesis of Fmoc-$_p$y. a. H_3PO_4, P_2O_5, H_2O; b. Fmoc-Osu, Triethylamine.

Briefly, phosphorus pentoxide (10.0 g) and 85% of phosphoric acid (13.0 g) were charged into a round flask under a nitrogen atmosphere. D-tyrosine (3.22 g) was

added, and the mixture was stirred and heated to 80 °C for 24 h. After completion of the reaction, water (30 mL) was added, and stirring was continued for an additional 30 min. The reaction mixture was then cooled to room temperature, diluted with n-butanol (650 mL), and stored at 4 °C overnight. The resulting white precipitate was collected by filtration, washed with ice-cold water, ethanol, and diethyl ether, and dried to afford a white powder, which was used in the next step without further purification.

To introduce the Fmoc group, phosphotyrosine (2.5 g), Fmoc-Osu (4.0 g), and triethylamine (adjusted to pH to 8~9) were suspended in a mixture of water (25 mL) and acetonitrile (25 mL). The reaction mixture was stirred at room temperature for 2 h. The organic solvent was then removed under reduced pressure, and ethyl acetate (300 mL) and an equal volume of water were added. The mixture was acidified to pH ~2 with HCl. The organic layer was separated, and the aqueous phase was extracted twice with ethyl acetate. The combined organic layers were washed with 1N HCl, H_2O, and brine, dried over anhydrous MgSO4, and concentrated under reduced pressure to afford Fmoc-$_p$y protected phosphotyrosine as white powder.

Synthesis of peptide derivatives (SPPS)

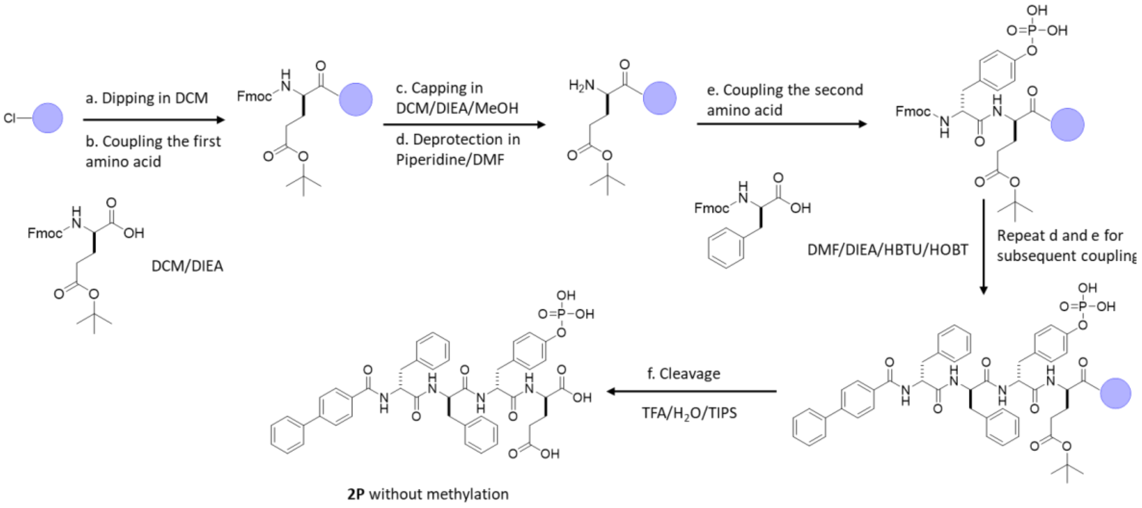

Scheme S2. The synthesis of peptides derivatives without methylation.

Following the synthesis of Fmoc-protected phosphotyrosine, peptide derivatives without methylation were prepared using standard Fmoc solid-phase peptide synthesis. This involves the use of 2-chlorotrityl chloride resin and the corresponding Fmoc-protected amino acids with appropriately protected side chains.

Solid-phase peptide synthesis (SPPS) was performed on 2-chlorotrityl chloride resin. The resin was swollen in dichloromethane (DCM) for 15 min prior to use. Fmoc-protected amino acids (1.2 mmol per gram of resin) were dissolved in DCM and activated with N,N-diisopropylethylamine (DIPEA, 2.5 equiv), then added to the resin and agitated on a rocker for 1 h. The resin was subsequently washed with DCM.

Unreacted sites were capped using a solution of DCM/MeOH/DIPEA (17:2:1, v/v/v) for 15 min, followed by washing with DCM and N,N-dimethylformamide (DMF). Fmoc deprotection was carried out using 20% piperidine in DMF for 30 min, followed by washing with DMF.

For peptide elongation, Fmoc-protected amino acids (1 equiv), HBTU (1 equiv), and DIPEA (2.5 equiv) in DMF were added to the resin and allowed to react for 40 min. The resin was washed with DMF after each coupling step. Residual DMF was removed by washing with DCM.

Final cleavage of the peptide from the resin was performed using a cleavage cocktail of trifluoroacetic acid (TFA, 95%), triisopropylsilane (TIPS, 2.5%), and water (2.5%) for 30 min. Collect the solution.

Synthesis of methylated peptide derivatives

After obtaining the peptide derivatives, the peptides (1 equiv) was dissolved in dichloromethane (DCM) with stirring. Bromotrimethylsilane (TMSBr, 15 equivalent) was then added, and the reaction mixture was stirred at room temperature overnight. The solvent was air dried, and methanol was subsequently added. The mixture was further stirred at room temperature for 24 h. The resulting product was purified with high-performance liquid chromatography (HPLC).

TEM sample preparation

Solution preparation

Peptides were initially dissolved in dimethyl sulfoxide (DMSO) to prepare stock solutions and subsequently diluted with phosphate-buffered saline (PBS). Stock solutions (200× the final concentration) were prepared in dimethyl sulfoxide (DMSO) and diluted with phosphate-buffered saline (PBS) to the desired working concentrations. All samples were prepared by adding appropriate volumes of stock solutions to PBS, ensuring a constant DMSO content (0.5% v/v). The same dilution procedure was applied to all other samples.

Grid preparation

Transmission electron microscopy (TEM) samples were prepared by negative staining. 5 μL aliquot of sample solution was applied onto a carbon-coated copper grid, and allowed to absorb for 30 s. Excess solution was removed by blotting with filter paper. The grid was the rinsed by briefly contacting it with drops of deionized water (ddH$_2$O) placed on parafilm, with the sample-loaded surface facing the droplet. Excess liquid was removed by blotting from the edge of the grid, and the rinsing step was repeated three times. This process was repeated three times.

Immediately after rinsing, the grid was stained by contacting it with a drop of uranyl acetate (UA) solution on parafilm. Excess stain was removed by blotting with filter paper. The grid was then air-dried and examined by TEM.

Critical micelle concentration (CMC) measurement

A series of precursor (**1P-15P**) solutions from the concentration of 2 mM to 0.2 μM was prepared in pH 7.4 PBS buffer. After incubating with Rhodamine 6G (5 μM), the λ_{max} was determined by measuring the absorbance from 520 to 540 nm using a Biotek Synergy 4 hybrid multi-mode microplate reader.

Cell culture

Saos-2, HeLa, MCF-7, HEK-293, Hep G2, and PANC-1 cell lines were purchased from ATCC. OVSAHO, Kuramochi and SK-OV-3 cell lines were obtained from Dr. Daniela M. Dinulescu. Saos-2 cell line was cultured in McCoy's 5A Medium (Gibco, Life Technologies) supplemented with 15% (v/v) fetal bovine serum (FBS) (Gibco, Life Technologies), 100 U/mL penicillin and 100 μg/mL streptomycin (Gibco, Life Technologies). HeLa, MCF-7, HEK-293 and Hep G2 cell lines were cultured in Minimum Essential Medium (Gibco, Life Technologies) supplemented with 10% (v/v) FBS, 100 U/mL penicillin and 100 μg/mL streptomycin. OVSAHO and Kuramochi cell lines were cultured in RPMI1640 (ATCC, USA) Medium supplemented with 10% (v/v) FBS, 100 U/mL penicillin and 100 μg/mL streptomycin SK-OV-3 cells were cultured in McCoy's 5A Medium (Gibco, Life Technologies) supplemented with 10% (v/v) fetal bovine serum (FBS) (Gibco, Life Technologies), 100 U/mL penicillin and 100 μg/mL streptomycin (Gibco, Life Technologies). PANC-1 cell line was cultured in Dulbecco's Modified Eagle's Medium (Gibco, Life Technologies) supplemented with 10% (v/v) fetal bovine serum (FBS) (Gibco, Life Technologies), 100 U/mL penicillin and 100 μg/mL streptomycin (Gibco, Life Technologies). All the cells were maintained at 37 °C in a humidified atmosphere of 5% CO_2.

MTT assay

Solution preparation

Compounds were initially dissolved in dimethyl sulfoxide (DMSO) to prepare stock solutions and subsequently diluted with cell culture medium to the desired working concentrations. Appropriate volumes of stock solutions were added to the culture medium to achieve the target compound concentrations while maintaining a constant DMSO content (0.5%). Control samples contained the same concentration of DMSO as the treated groups.

5 μL stock solution (mM)	995 μL culture medium	Final concentration (μM)
10	995	50
4	995	20
2	995	10
1	995	5
0.4	995	2

Cells were seeded in 96-well plates at a density of 1×10^4 cells per well and incubated for 24 h to allow attachment. The culture medium was then replaced with fresh culture medium containing the precursors at desired concentrations. After incubation for 24, 48, or 72 h, MTT solution (10 μL, 5 mg/mL in PBS) was added to each well, and the plates were incubated at 37 °C for 4 h. Subsequently, SDS-HCl solution (100 μL) was added to each well to terminate the reaction and dissolve the formazan crystals.

The absorbance at 595 nm was measured using a DTX880 Multimode Detector. Cell viability was calculated as a percentage relative to untreated control cells. All experiments were performed in triplicate (n = 3), and data are presented as the mean of three independent measurements.

Supplementary Figures

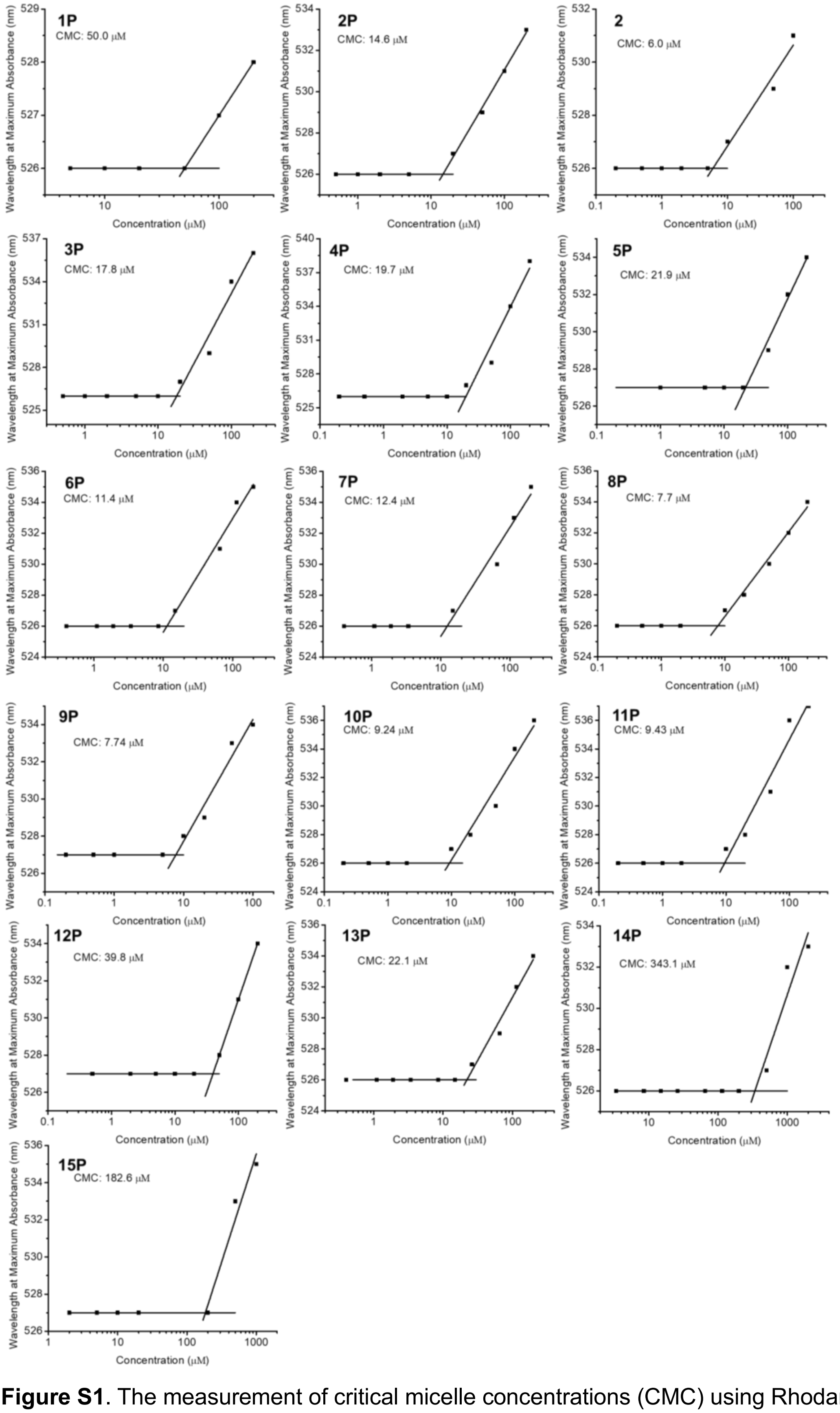

Figure S1. The measurement of critical micelle concentrations (CMC) using Rhodamine 6G.

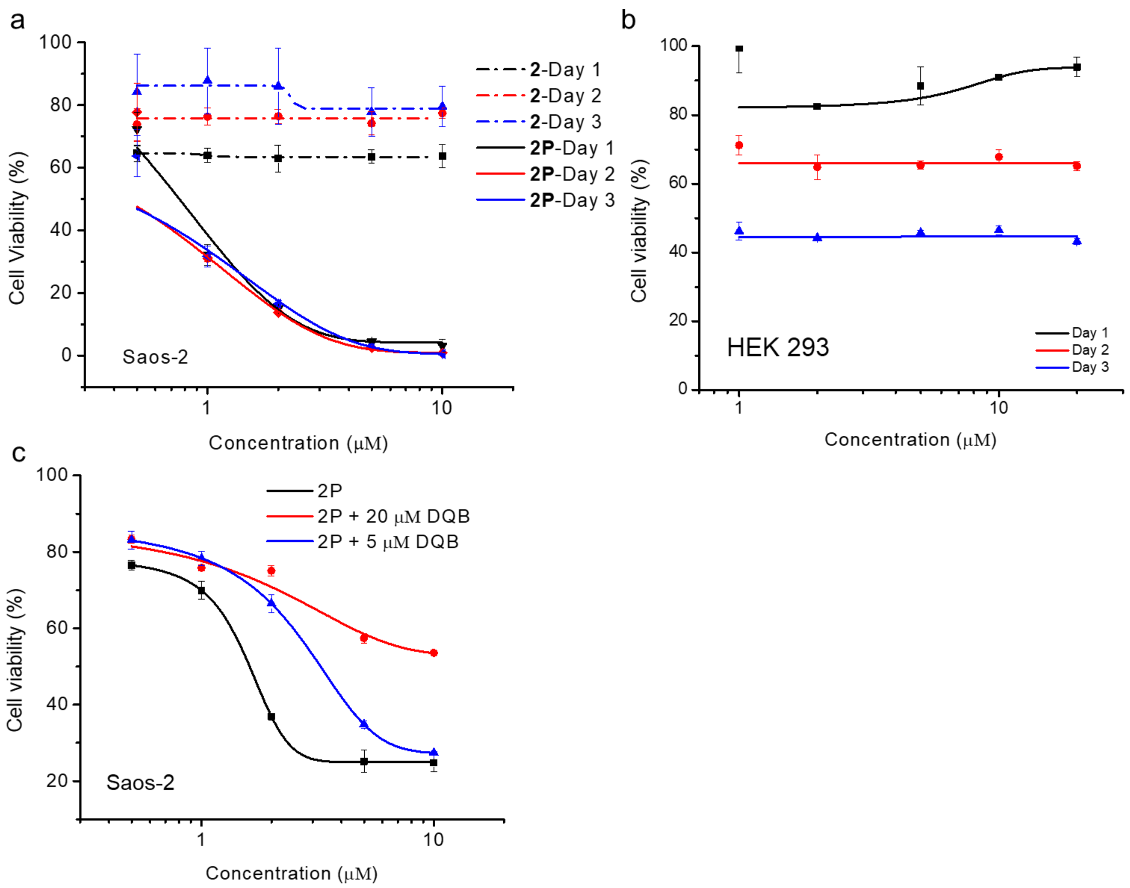

Figure S2. The role of phosphate on EISA. a. The cell viability of Saos-2 cells treated with 2 or 2P for 1 day, 2 days, or 3 days. b. The cell viability of HEK-293 cells treated with 2P for 1 day, 2days, or 3 days. c. The cell viability of Saos-2 cells treated with 2P, 2P cocultured with 20 μM DQB or 5 μM DQB for 1 day.

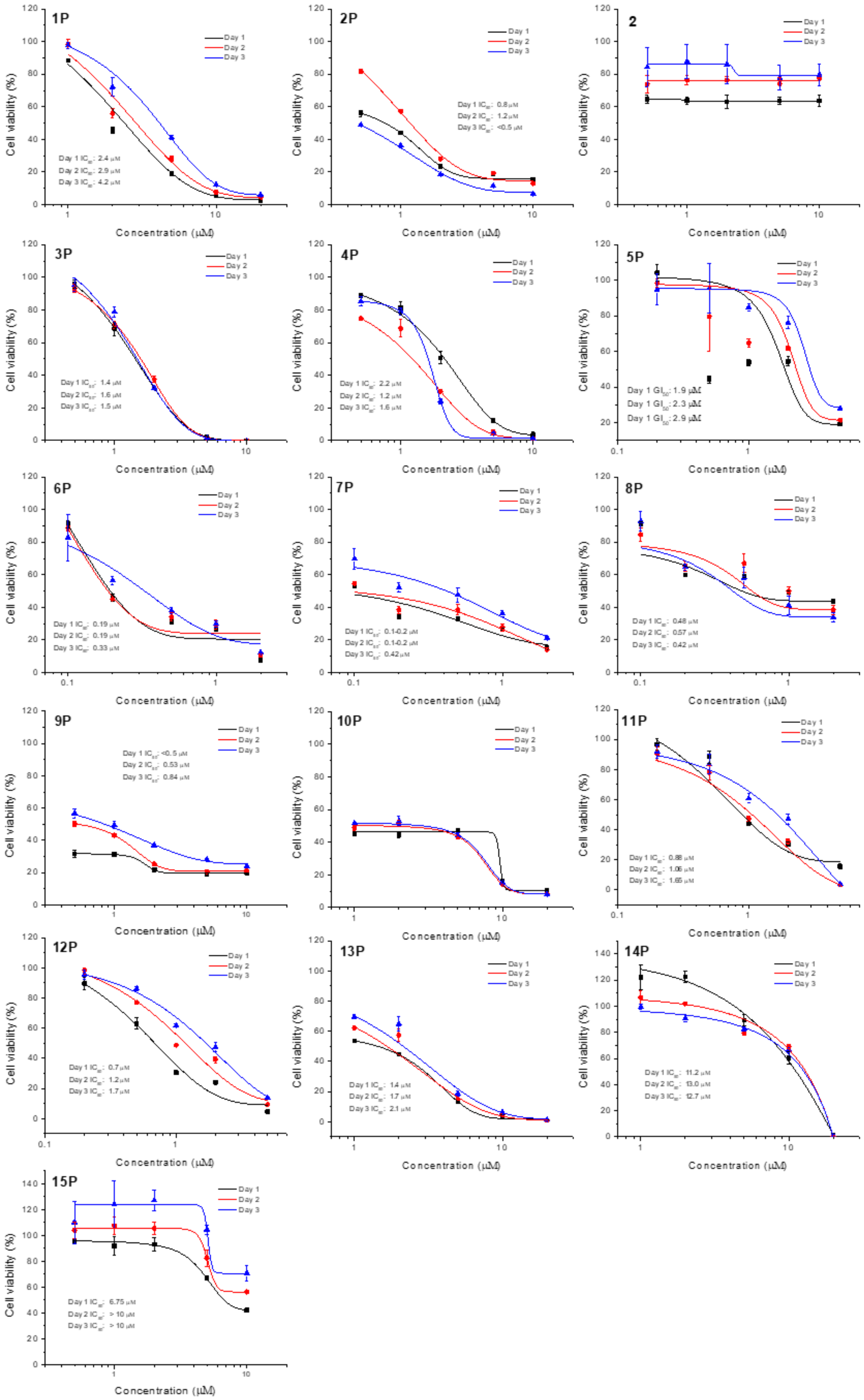

Figure S3. The cell viability of Saos-2 cells treated with different EISA precursors.

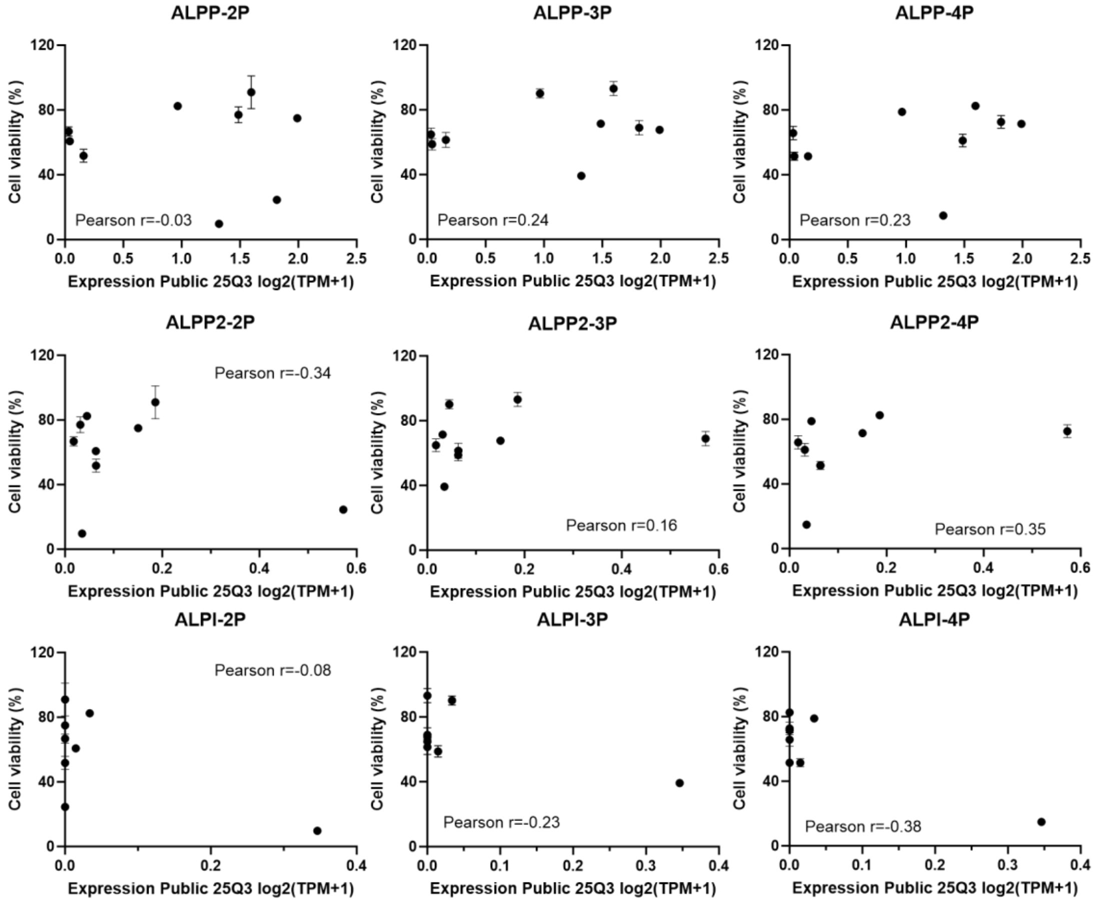

Figure S4. The relationship between gene expression and cell inhibition. The cell viability of different cell lines at 2 μM for 24 h vs ALPP, ALPP2, and ALPI gene expression levels according to Depmap CCLE.

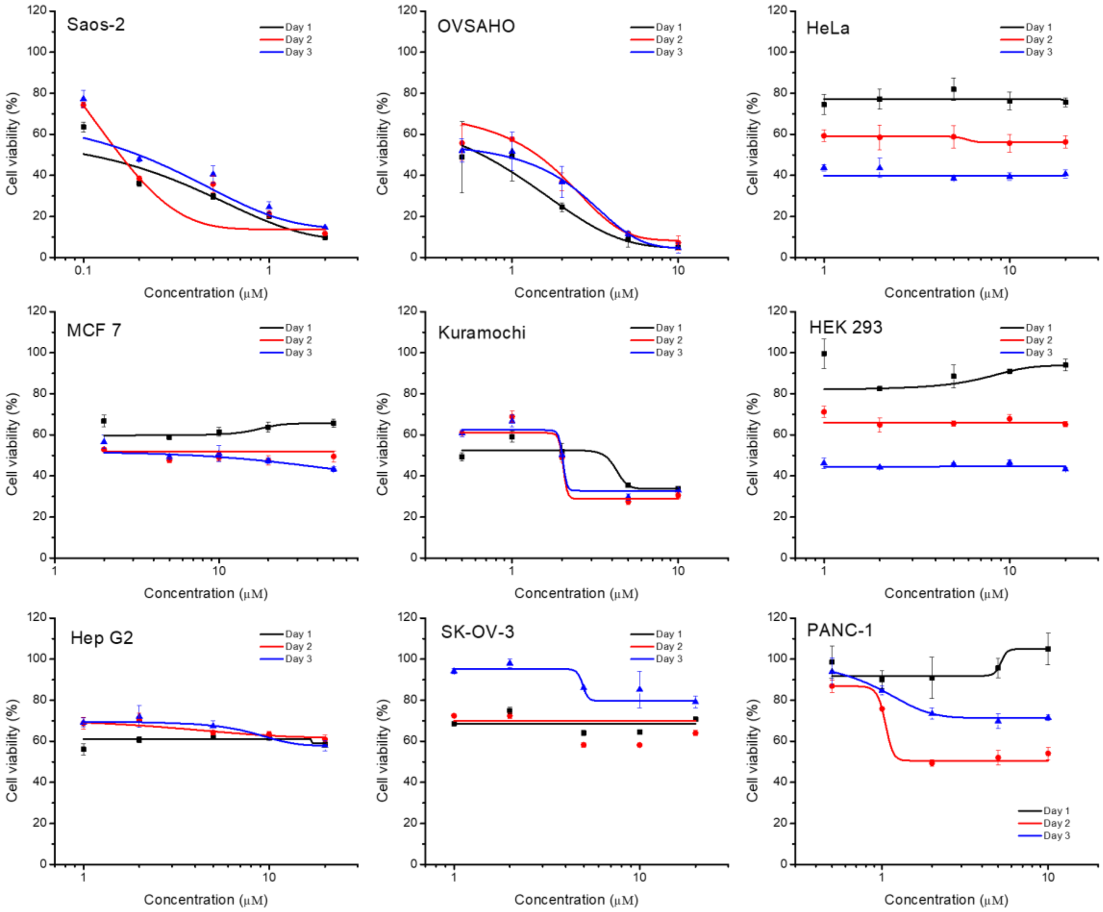

Figure S5. The cell viability of Saos-2, OVSAHO, HeLa, MCF-7, Kuramochi, HEK-293, Hep-G2, SK-OV-3, and PANC-1 treated with **2P**.

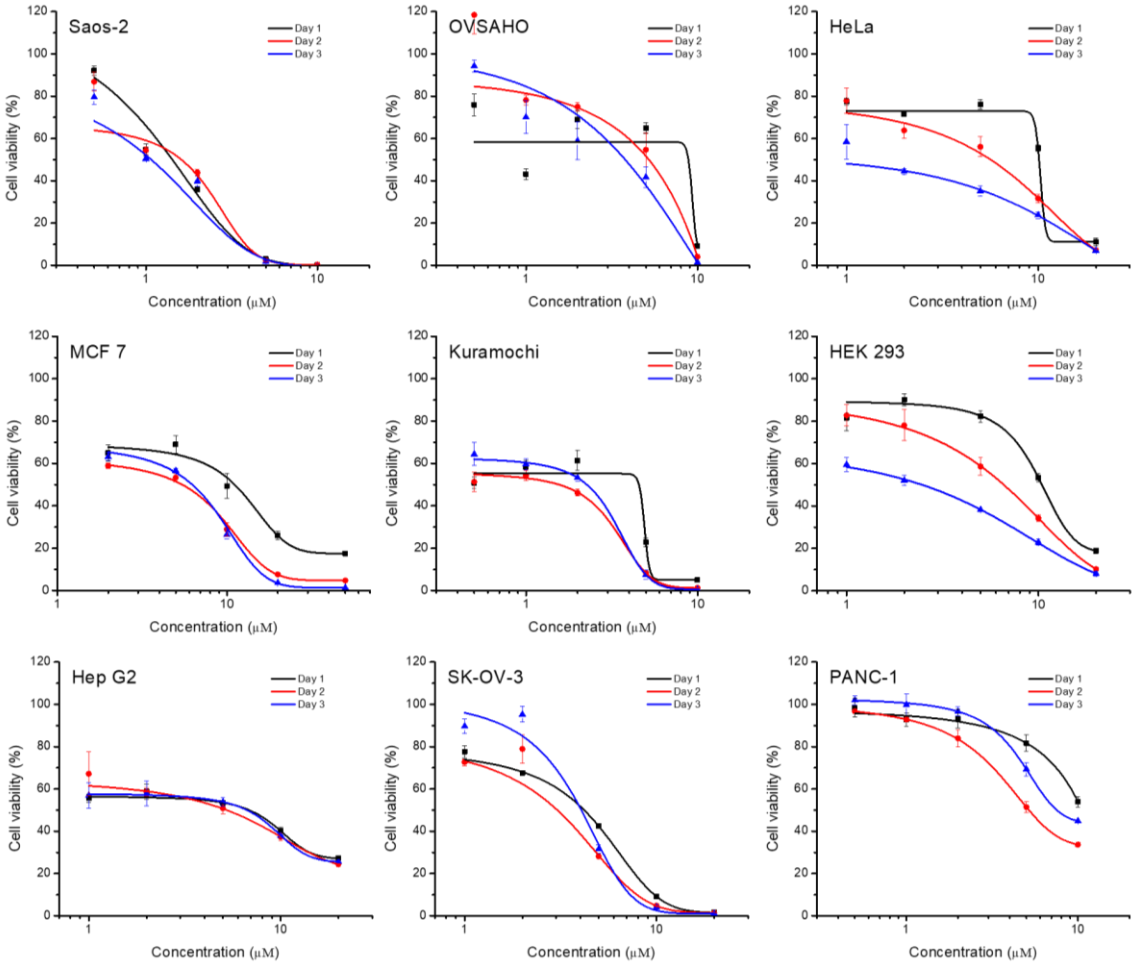

Figure S6. The cell viability of Saos-2, OVSAHO, HeLa, MCF 7, Kuramochi, HEK-293, Hep G2, SK-OV-3, and PANC-1 treated with **3P**.

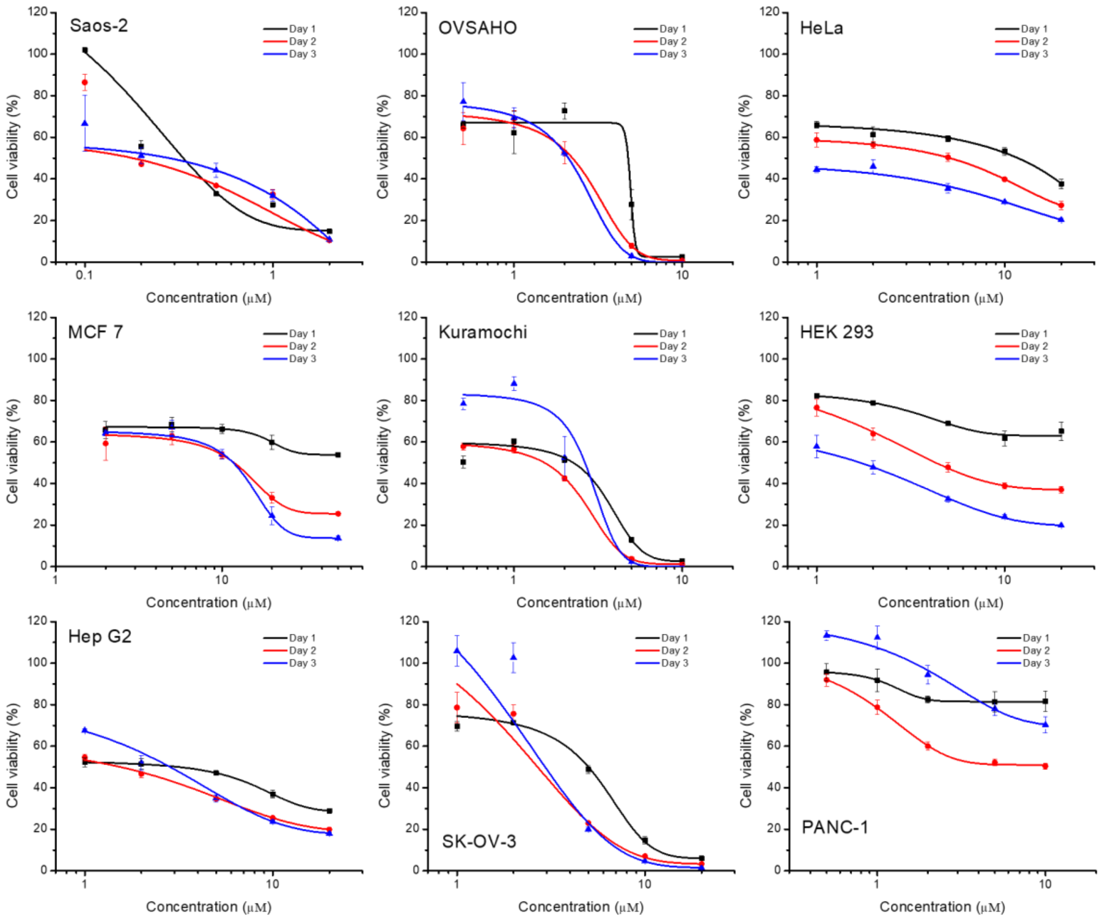

Figure S7. The cell viability of Saos-2, OVSAHO, HeLa, MCF 7, Kuramochi, HEK-293, Hep G2, SK-OV-3, and PANC-1 treated with **4P**.

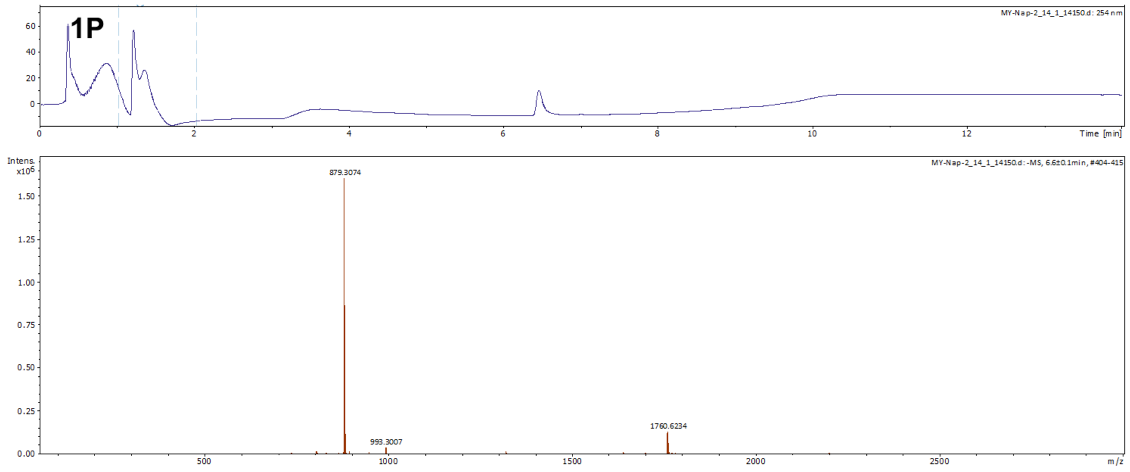

Figure S8. The LCMS of **1P**.

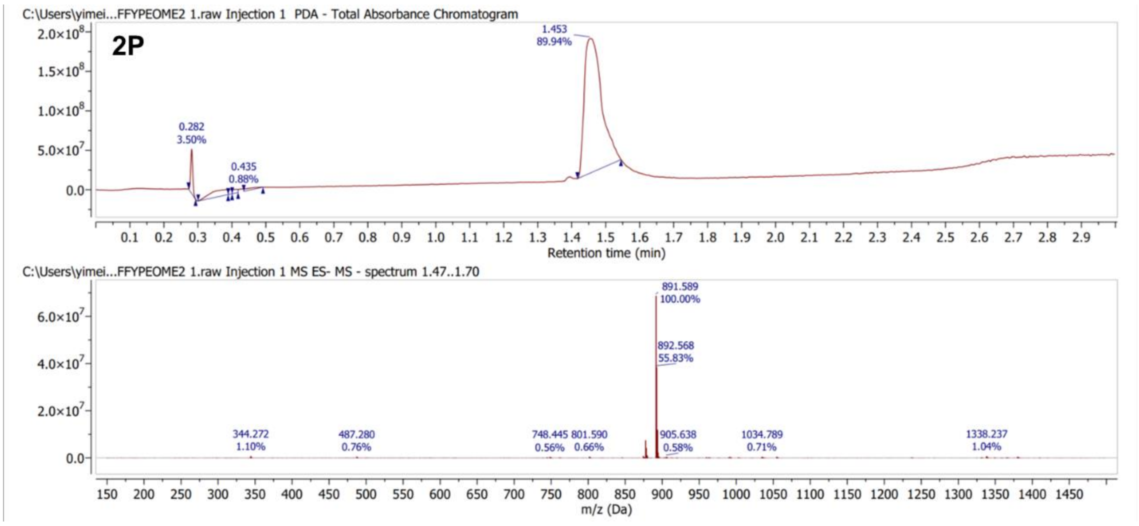

Figure S9. The LCMS of **2P**.

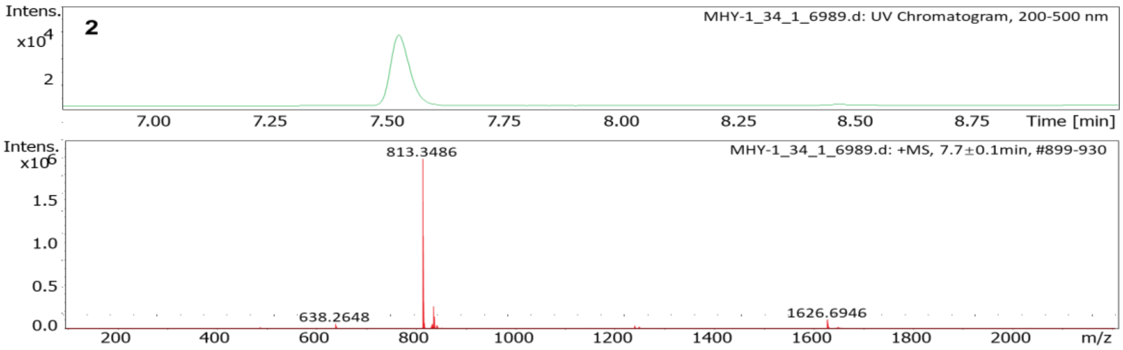

Figure S10. The LCMS of **2**.

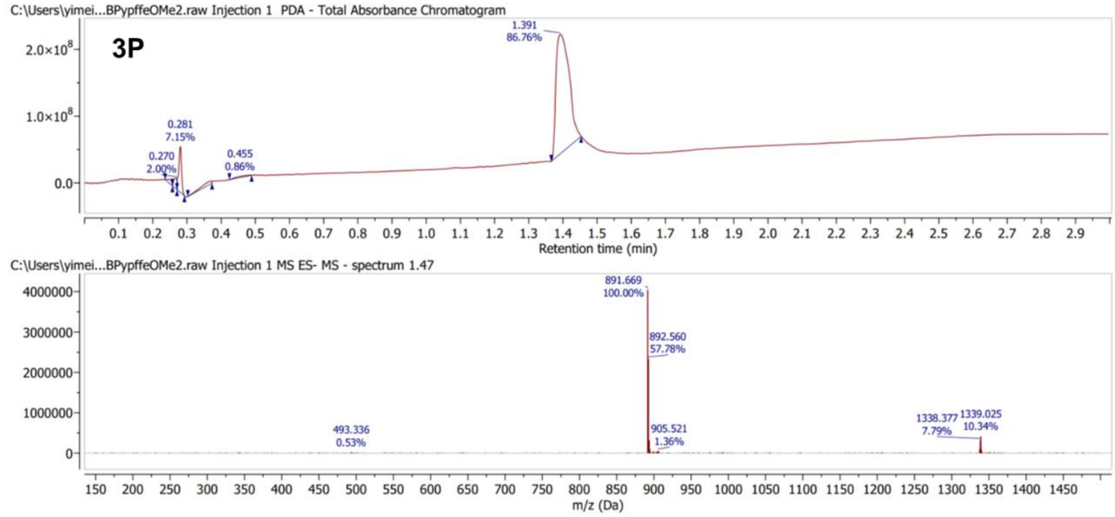

Figure S11. The LCMS of **3P**.

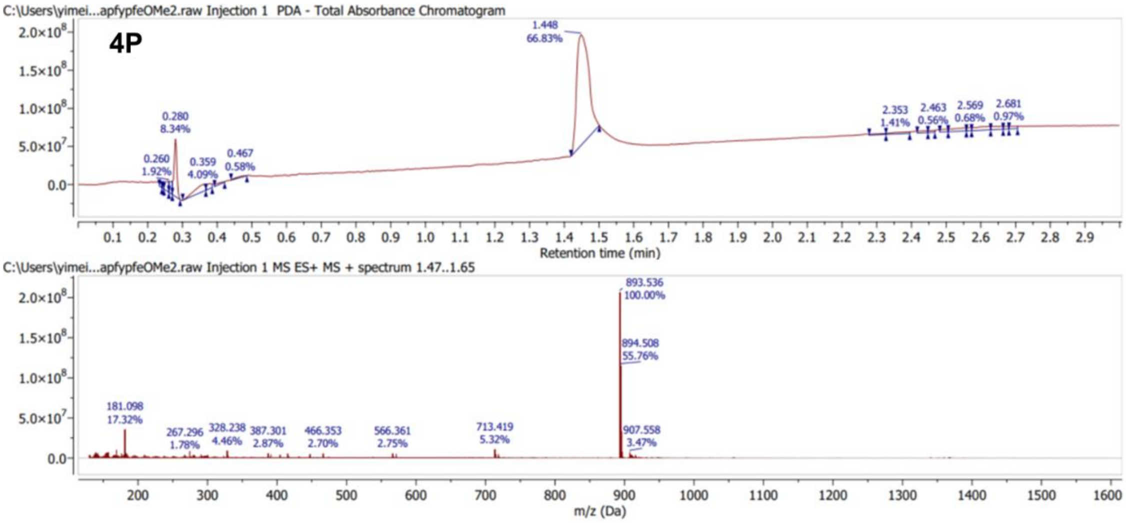

Figure S12. The LCMS of **4P**.

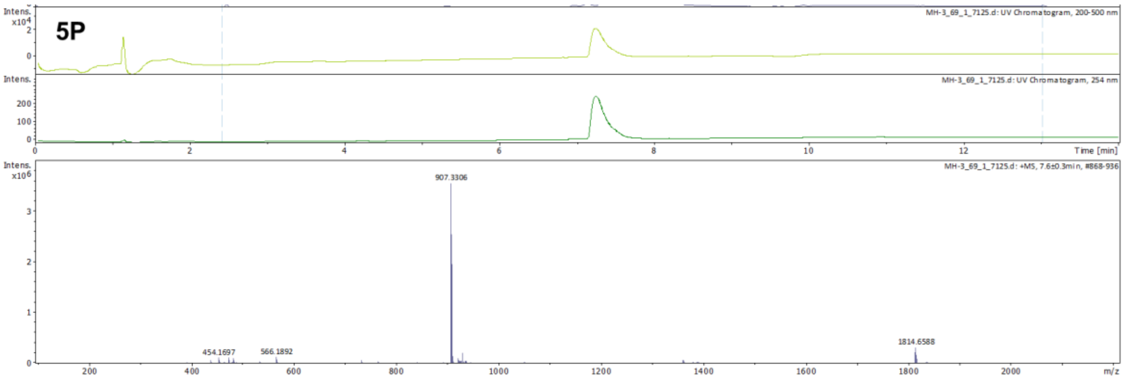

Figure S13. The LCMS of **5P**.

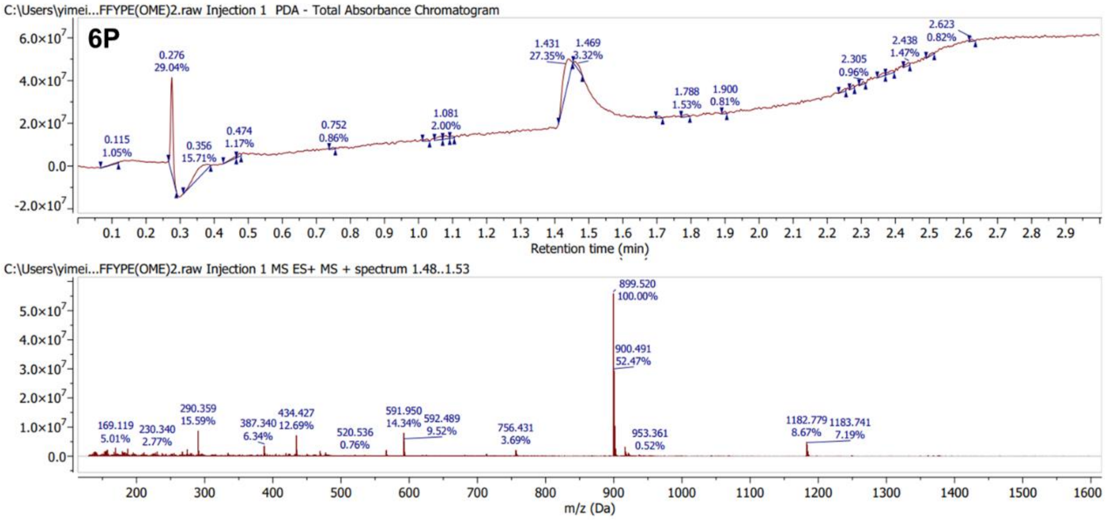

Figure S14. The LCMS of **6P**.

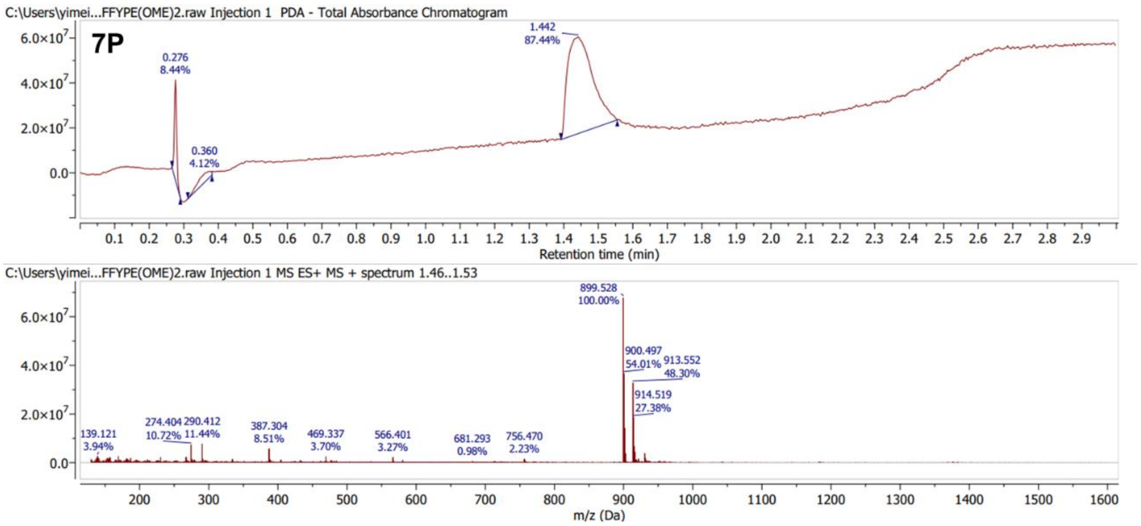

Figure S15. The LCMS of **7P**.

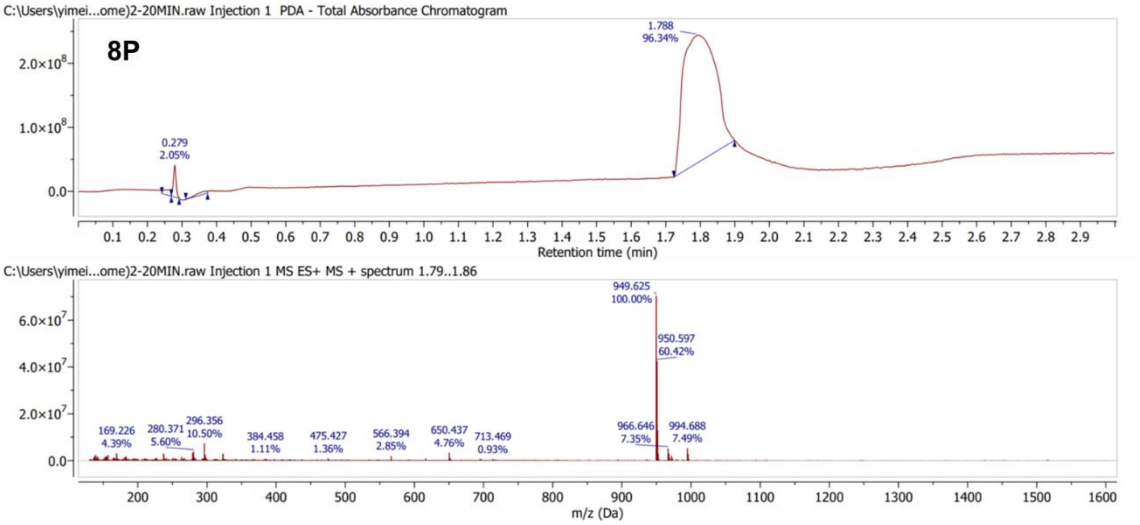

Figure S16. The LCMS of **8P**.

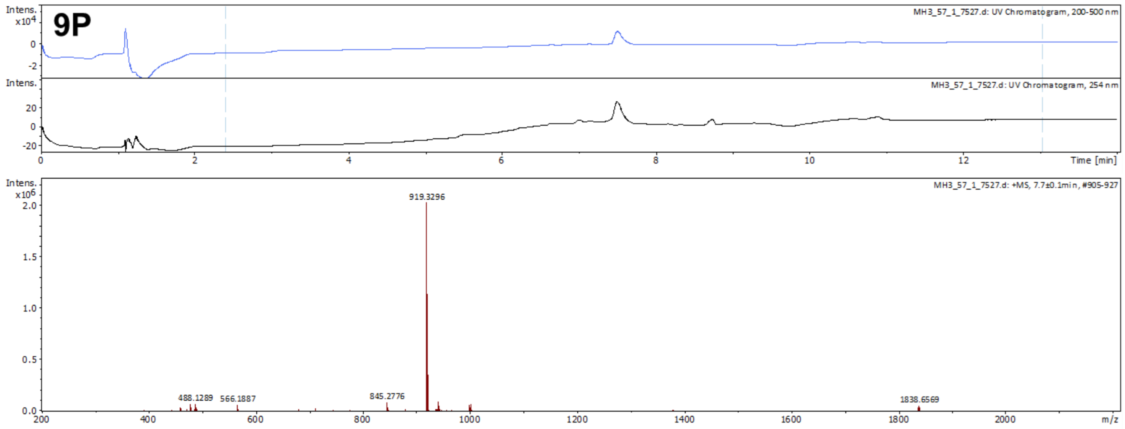

Figure S17. The LCMS of **9P**.

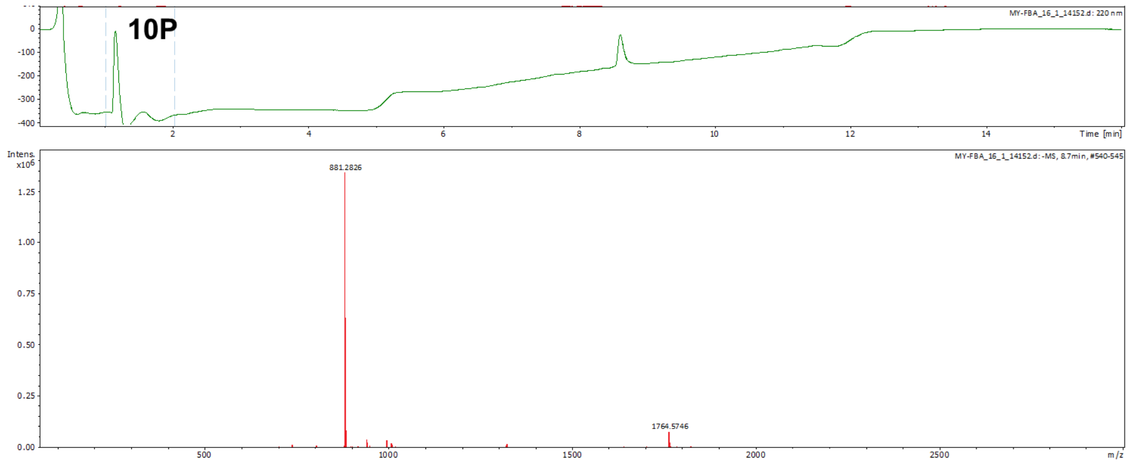

Figure S18. The LCMS of **10P**.

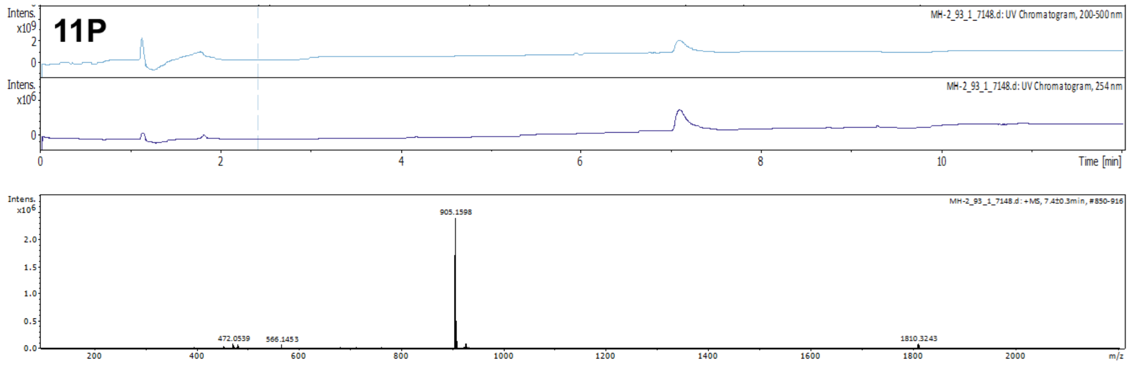

Figure S19. The LCMS of **11P**.

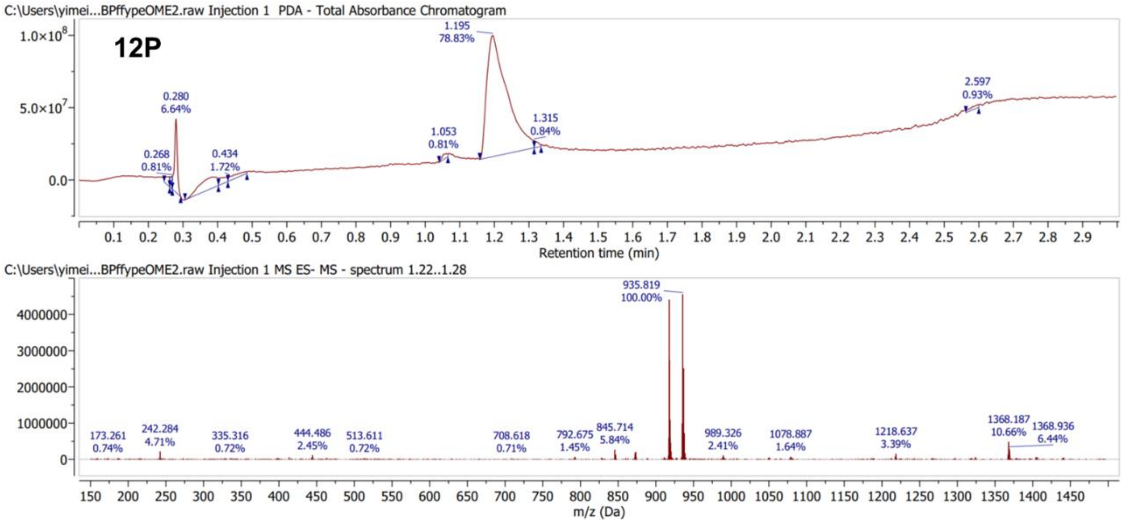

Figure S20. The LCMS of **12P**.

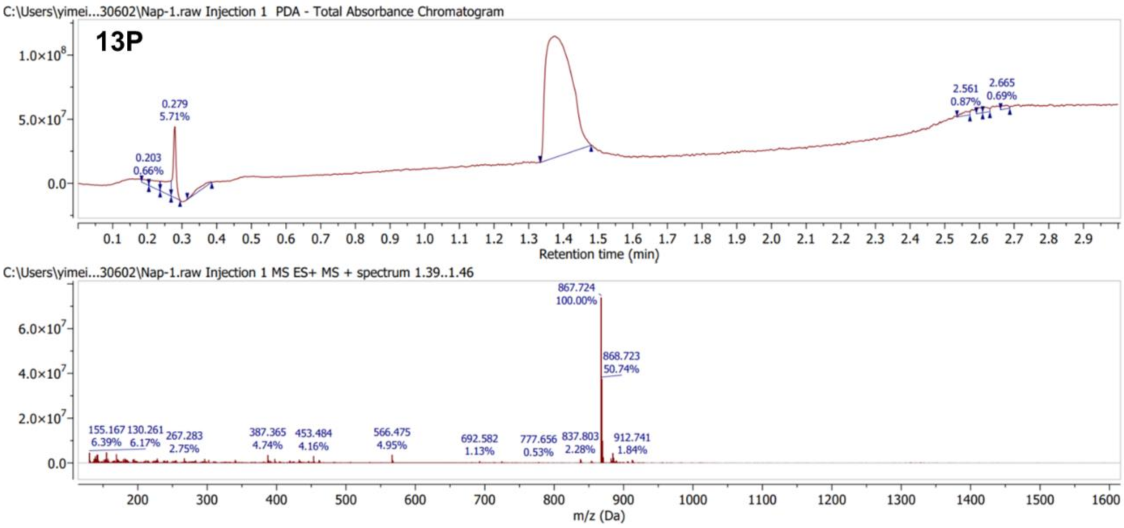

Figure S21. The LCMS of **13P**.

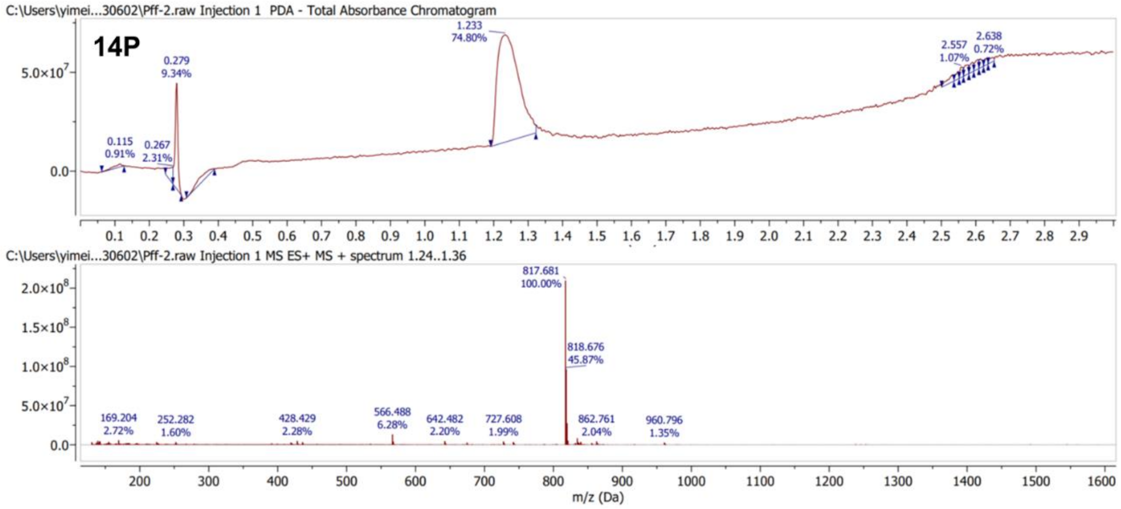

Figure S22. The LCMS of **14P**.

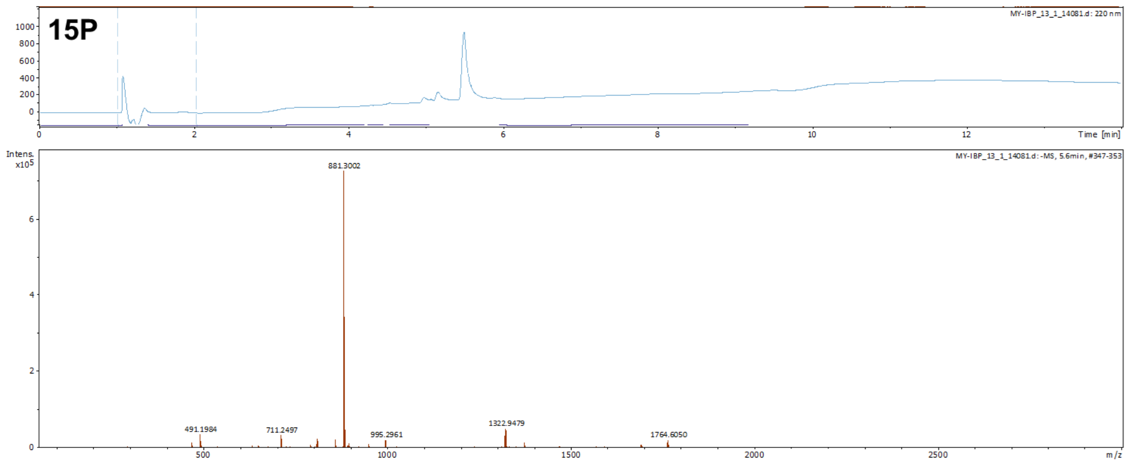

Figure S23. The LCMS of **15P**.
